# Monitoring the action of redox-directed cancer therapeutics using a human peroxiredoxin-2-based probe

**DOI:** 10.1038/s41467-018-05557-y

**Published:** 2018-08-07

**Authors:** Troy F. Langford, Beijing K. Huang, Joseph B. Lim, Sun Jin Moon, Hadley D. Sikes

**Affiliations:** 1 Department of Chemical Engineering, Massachusetts Institute of Technology, 77 Massachusetts Avenue, Cambridge, 02139 USA; 2 Department of Biological Engineering, Massachusetts Institute of Technology, 77 Massachusetts Avenue, Cambridge, 02139 USA

## Abstract

Redox cancer therapeutics target the increased reliance on intracellular antioxidant systems and enhanced susceptibility to oxidant-induced stress of some cancer cells compared to normal cells. Many of these therapeutics are thought to perturb intracellular levels of the oxidant hydrogen peroxide (H_2_O_2_), a signaling molecule that modulates a number of different processes in human cells. However, fluorescent probes for this species remain limited in their ability to detect the small perturbations induced during successful treatments. We report a fluorescent sensor based upon human peroxiredoxin-2, which acts as the natural indicator of small H_2_O_2_ fluctuations in human cells. The new probe reveals peroxide-induced oxidation in human cells below the detection limit of current probes, as well as peroxiredoxin-2 oxidation caused by two different redox cancer therapeutics in living cells. This capability will be useful in elucidating the mechanism of current redox-based therapeutics and in developing new ones.

## Abstract (web summary)

Current sensors for intracellular H_2_O_2_ are not sensitive enough to detect all biologically relevant H_2_O_2_ fluctuations. Here the authors develop a peroxiredoxin-2-based FRET probe that is sensitive enough to measure changes in H_2_O_2_ concentration in response to the cancer therapeutic piperlongumine.

## Introduction

Hydrogen peroxide (H_2_O_2_) can regulate a variety cellular processes via the oxidation of specific cysteine residues of redox-regulated proteins, which can trigger a range of cellular responses, including cell growth and proliferation at lower levels of H_2_O_2_, as well as autophagy, apoptosis, and necrosis at higher levels of this oxidant^1–5^. Many cancer cells are thought to have much higher rates of production of H_2_O_2_ compared to normal cells due to a combination of both aberrant metabolism and genetic mutations^6,7^. As a result of this additional H_2_O_2_ production, many of these cells are thought to have higher susceptibility to H_2_O_2_-induced apoptosis compared to normal cells^8–12^. In order to combat this increase in oxidant production, cancer cells also upregulate key antioxidant species in order to remove this oxidant from the cell and ensure their survival^6,8^. Redox cancer therapeutics target cancer cells’ increased dependence on intracellular antioxidant systems and enhanced susceptibility to H_2_O_2_-induced stress through inhibition of these antioxidant systems that detoxify the cell, resulting in elevation of oxidants within the cell^8–12^. Several small molecule cancer therapeutics, such as piperlongumine^13^, are thought to result in elevated intracellular H_2_O_2_ that is central to their toxicity mechanisms; however, due to the small perturbations produced in response to several of these drugs, it remains difficult to detect H_2_O_2_ fluctuations in response to these treatments^14^.

In human cells, cytosolic H_2_O_2_ levels are controlled by a powerful network of specific reductive reactions primarily accomplished by peroxiredoxins (Prxs), thioredoxin (Trx), and thioredoxin reductase (TrxR), with reducing equivalents supplied by nicotinamide adenine dinucleotide phosphate (NADPH)^15,16^. Winterbourn et al.^2^ noted that human Prxs should scavenge the majority of H_2_O_2_ in human cells on the basis of their second-order rate coefficients for reaction with H_2_O_2_ (on the order of 1 × 10^7^ M^−1^s^−1^)^17^ and abundance in the cytosol of mammalian cells (on the order of 1 × 10^−4^ M)^16,18^. Further analysis of this pathway with detailed kinetic models revealed that H_2_O_2_ clearance models that only consider H_2_O_2_ consumption by Prxs and neglect all other antioxidants produce the same clearance curves for all reasonable levels of H_2_O_2_ as those predicted by a full metabolic model of H_2_O_2_ clearance^16^. One abundant isoform, Prx2, achieves this high reaction rate via the exceptionally reactive thiol group of a cysteine residue near the N-terminus of the protein known as the catalytic cysteine^19,20^. When the catalytic cysteine is oxidized to a sulfenic acid, it forms a disulfide bond with a second cysteine residue, known as the resolving cysteine, of an adjacent Prx2 monomer. This process involves a conformational change and results in a covalently-linked dimer species. After dimer formation, Trx reduces the disulfide bond between the two Prx2 monomers and regenerates the original proteins, while TrxR reduces oxidized Trx using electrons from NADPH^19,20^. This set of reactions maintains a large amount of reduced Prx2 in the cytosol of cells^16^, poised to respond to very small changes in cytosolic H_2_O_2_ fluxes. This feature of the system suggests that Prx2 oxidation could serve as an indicator for fluctuations in physiological levels of H_2_O_2_, and that human Prx2 fused with one or more fluorescent proteins could serve as the basis of a specific and sensitive sensor for H_2_O_2_.

Currently, the most common way to measure H_2_O_2_-induced Prx2 oxidation is through non-reducing gel-electrophoresis followed by western blot for Prx2 monomers and dimers^21^. While this approach has proven useful in many studies, it requires laborious gel electrophoresis procedures, pooled cell lysate samples, and often results in a large amount of variability from sample to sample. For ultrasensitive H_2_O_2_ detection, several groups have designed genetically-encoded fluorescent probes for H_2_O_2_-induced peroxiredoxin oxidation; this approach has yielded success in yeast^22^, where the designed probe was more sensitive than the current genetically-encoded sensors HyPer and roGFP2-Orp1, as well as plants^23–25^, but has not been applied to human cells, where HyPer and roGFP2-Orp1 are state-of-the-art tools. In this work, we created a fluorescence resonance energy transfer (FRET) probe based upon the reactivity of human Prx2 for ultrasensitive detection of H_2_O_2_ in human cells. In order to maximize the fluorescence change in the two fluorophores upon dimerization of the reactive domain of the probe, we chose the fluorescent proteins Clover and mRuby2 as a FRET pair, which were previously shown to have a higher dynamic range than conventional cerulean-yellow fluorescent protein FRET pairs and insensitivity to changes in pH^26^. In order to validate this new tool, we purified the fluorescent probe and quantified its fluorescent signal in response to various oxidants and buffers in order to determine its selectivity and pH sensitivity. Additionally, we expressed the probe in human epithelial cells and quantified its fluorescent signal in response to various perturbations chosen to assess sensitivity towards H_2_O_2_. Following these detailed characterization and validation studies, we were able to detect and track low levels of H_2_O_2_-induced Prx2 oxidation when malignant cells were treated with the cancer therapeutics auranofin and piperlongumine. Even though the small elevation of H_2_O_2_ in response to piperlongumine treatment was sufficient to cause apoptosis^14^, the H_2_O_2_ level was below the lower detection limit of HyPer, which highlights the unique ability of the probe developed in this work to detect small, yet relevant, changes in intracellular H_2_O_2_ levels using a responsive domain that is of broad interest in many biological processes^27–29^.

## Results

### Probe responds rapidly and robustly to H_2_O_2_

**Fig. 1:**
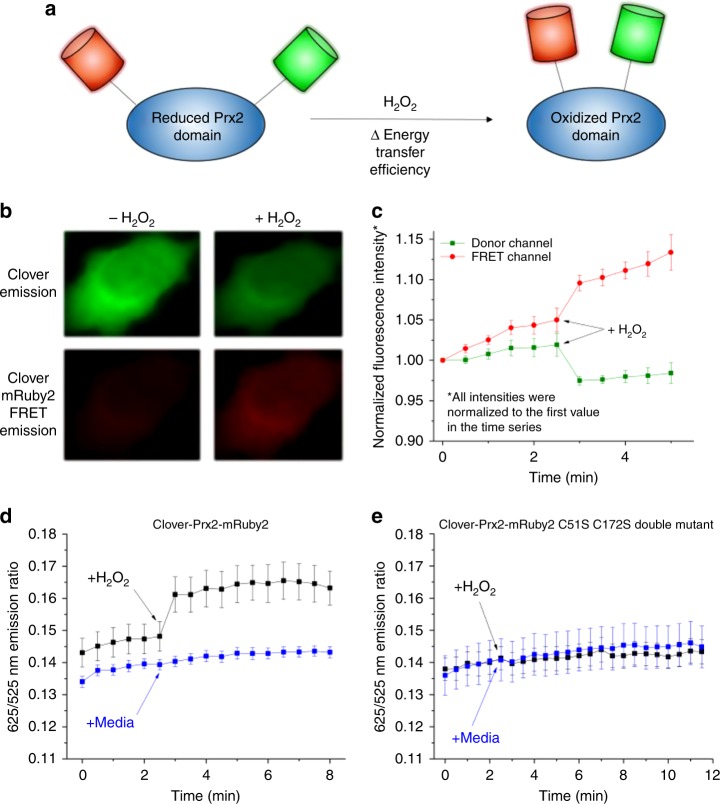
Design and function of human Prx2-based FRET probe. **a** Schematic representation of Prx2 based probe. **b** Response of cytosolic sensor upon oxidation by H_2_O_2_. Changes in each of the two fluorescent channels upon stimulation with H_2_O_2_ suggest that the fusion protein adopts a conformation in which the two fluorophores are in closer proximity in the oxidized state. Pseudo-coloring of one HeLa cell is shown for illustrative purposes. **c** Normalized emission from the donor and FRET fluorescent channels over time upon stimulation with approximately 60 × 10^−15^ mol per cell in HeLa cells expressing the functional Prx2 probe. **d** 625/525 nm emission ratio over time in HeLa cells expressing the functional Prx2 probe upon stimulation with a control bolus (culture medium only, blue line) or stimulation with approximately 60 × 10^−15^ mol H_2_O_2_ per cell (black line). **e** 625/525 nm emission ratio over time in HeLa cells expressing Prx2-based probe with both the Prx2 catalytic cysteine residue and resolving cysteine residue mutated to serine upon stimulation with a control bolus (culture medium only, blue line) or stimulation with approximately 60 × 10^−15^ mol H_2_O_2_ per cell (black line). 2 mL of 20 μM H_2_O_2_ was added to 7 × 10^5^ adherent cells to achieve 60 × 10^−15^ mol H_2_O_2_ per cell. Data points represent mean emission ratio ± S.E.M. for nine cells and are reflective of three biological replicates

In order to construct an ultrasensitive H_2_O_2_ probe, the genes corresponding to the three domains of the probe (Clover, Prx2, mRuby2) were inserted into pcDNA3.1+ via the Gibson assembly method. Additionally, between each fluorescent protein and the central Prx2 domain, a short flexible amino acid linker was inserted to promote proper folding of the fluorescent protein domains (Fig. 1a). In order to characterize the response of the cytosolic probe to H_2_O_2_, a culture of HeLa cells was transfected with the gene encoding the probe, exposed to an external bolus addition of H_2_O_2_, and imaged with a widefield microscope. Upon addition of H_2_O_2_ to the culture, the intensity in the donor fluorescence channel decreased while the intensity in the donor–acceptor FRET fluorescence channel increased (Fig. 1b, c). These changes resulted in a rapid increase in the emission ratio of the probe, defined as the ratio of intensity in the FRET fluorescence channel divided by the intensity in the donor fluorescence channel (Fig. 1d). Upon mutation of both the catalytic and resolving cysteine residues of the Prx2 domain of the probe to serine, addition of H_2_O_2_ did not elicit a change in the emission ratio, suggesting that the observed change in the signal of the unmodified probe depends on the activity of one, or both, of these cysteines (Fig. 1e). In contrast, mutation of only the catalytic cysteine of the probe followed by addition of H_2_O_2_ resulted in a slight increase in the emission ratio of the probe, but not as large as the original construct (Supplementary Fig. 1a), which suggests that disulfide bond linkages may also form between probe molecules and unmodified, endogenous Prx2 monomers that result in slight conformational changes in the probe. Interestingly, mutation of only the resolving cysteine of the probe followed by addition of H_2_O_2_ slightly decreased the emission ratio of the probe relative to the pre-stimulation value (Supplementary Fig. 1b), which may reflect hyperoxidation of the catalytic cysteine of the probe from a sulfenic acid to a sulfinic or sulfonic acid^30^. We present the results of these studies in terms of moles of H_2_O_2_ per cell as opposed to concentration or total moles of H_2_O_2_. As we and others have previously shown^31–34^, both the total moles of H_2_O_2_ added as well as the total number of cells present affect the results obtained from this kind of experiment, whereas moles of H_2_O_2_ per cell is a meaningful metric that may be compared across studies.

As a complement to intracellular measurements, we purified the fluorescent probe in order to assess its specificity to H_2_O_2_ and sensitivity to environmental factors such as pH. In order to purify the fluorescent protein, an N-terminal histidine-tagged construct was expressed in bacteria, and the recombinant protein was purified via immobilized metal affinity chromatography (Supplementary Fig. 2). The purified protein was subjected to various oxidants to determine the specificity of the probe (Supplementary Fig. 3 and Supplementary Fig. 4). Of the oxidants tested, only H_2_O_2_ and tert-butyl hydroperoxide (TBHP) significantly increased the emission ratio of the fluorescent protein, consistent with previous measurements with fluorescent peroxiredoxin-based constructs^22^. In addition to these experiments, the probe was also subjected to two different treatments to determine the sensitivity of peroxynitrite; in the first experiment, purified protein was exposed to the peroxynitrite generator compound linsidomine (SIN-1), whereas in the second, HeLa cells expressing the probe were exposed to a solution with nitric oxide, which diffuses into cells and rapidly reacts with superoxide to form peroxynitrite (Supplementary Fig. 5 and Supplementary Fig. 6). In both instances, the signal from the probe did not increase significantly after the treatment, once again consistent with previous measurements with analogous constructs^22^. In a separate set of experiments, we also tested the robustness of the purified probe to buffers with different pHs (Supplementary Fig. 7). The results of this study indicate that the emission ratio of the both of the reduced and oxidized probe remain relatively constant within the pH range of 6.6 to 7.8.

### Probe responds to low levels of extracellular H_2_O_2_

**Fig. 2:**
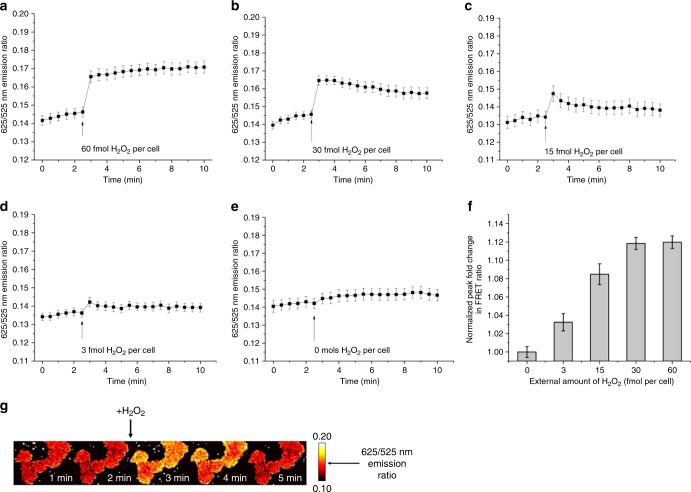
Concentration-dependent kinetics and magnitude of the fluorescent response to stimulation with H_2_O_2_. **a**–**e** 625/525 nm emission ratio as a function of time in HeLa cells expressing the Prx2-based probe upon stimulation with approximately 60 × 10^−15^ mol H_2_O_2_ per cell (**a**), 30 × 10^−15^ mol H_2_O_2_ per cell (**b**), 15 × 10^−15^ mol H_2_O_2_ per cell (**c**), 3 × 10^−15^ mol H_2_O_2_ per cell (**d**), and 0 × 10^−15^ mol H_2_O_2_ per cell (**e**). Mol per cell values correspond to 2 mL of 20, 10, 5, 1, or 0 μM H_2_O_2_ added extracellularly to 7 × 10^5^ adherent cells. **f** Normalized peak fold-change of the probe’s signal upon stimulation with different amounts of H_2_O_2_. Bar graph plus dot plot shown in Supplementary Fig. 23. Data points represent mean ratio ± S.E.M. for 18 cells from two biological replicates. **g** Probe ratio over time in several HeLa cells upon stimulation with the same amount of H_2_O_2_ as in part (**c**). Numbers next to the color scale represent raw emission ratios and the time points indicate time since start of image acquisition. H_2_O_2_ was added 2.5 min after the start of image acquisition. Dashed white lines represent boundary of each image

We further assessed the dynamics of probe oxidation and reduction with several additional H_2_O_2_ bolus perturbations. We first determined the amount of H_2_O_2_ required to completely saturate the probe (Fig. 2a), and then titrated down with lower quantities of H_2_O_2_ in order to study the dynamic behavior of the system (Fig. 2b-g, Supplementary Fig. 8). In order to test whether the probe responded to successive additions of H_2_O_2_, we also stimulated cells expressing the probe with a H_2_O_2_ bolus equivalent to that in Fig. 2c, waited until the emission ratio returned to the baseline level, and added a second H_2_O_2_ bolus of the same amount (Supplementary Fig. 9). In addition, in order to determine how long the probe signal persisted in the cell after saturation of the probe, we stimulated cells expressing the probe with a H_2_O_2_ bolus equivalent to that in Fig. 2a and waited until the emission ratio returned to the baseline level (Supplementary Fig. 1,0). Lastly, in order to determine the minimum and maximum fold changes for the cytosolic probe, cells with the probe were exposed to the thiol oxidant diamide and the disulfide reductase dithiothreitol, and the final fold change of the probe signal over the 10-min measurement period was calculated (Supplementary Fig. 1,1). As expected, maximum fold change achieved with diamide was higher than that achieved with H_2_O_2_ bolus addition, as diamide likely oxidizes intracellular reductases such as Trx and thus prevents the reduction of oxidized Prx protein. Additionally, the sensor fold change did not change upon addition of dithiothreitol to the cell culture, which suggests that the probe exists almost entirely in the reduced form at basal levels of intracellular H_2_O_2_. This finding agrees well with results from kinetic models of this system, which predict that approximately 99.8% of Prx2 resides in the reduced state at basal H_2_O_2_ concentrations^16^.

**Fig. 3:**
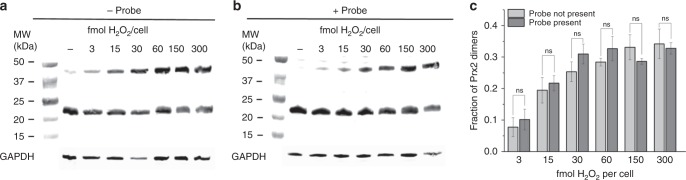
Effect of fluorescent Prx2 probe on H_2_O_2_ peroxidase activity of endogenous Prx2 in the cytosol of HeLa cells. **a** Endogenous Prx2 oxidation in HeLa cells without the fluorescent probe in response to various amounts of H_2_O_2_. **b** Endogenous Prx2 oxidation in HeLa cells stably expressing the probe in response to the same amounts of H_2_O_2_. Mol per cell values correspond to 2 mL of 20, 10, 5, 1, or 0 μM H_2_O_2_ added extracellularly to 7 × 10^5^ adherent cells. Endogenous glyceraldehyde 3-phosphate dehydrogenase (GAPDH) was simultaneously analyzed in these blots to assess the uniformity of lysate loading and protein content. Western blot images are reflective of three biological replicates. **c** Calculated fraction of dimeric Prx2 in cytosol of HeLa cells in response to listed amounts of H_2_O_2_. For each amount of H_2_O_2_, the fraction of dimeric Prx2 was calculated from the intensity of the dimer band divided by the sum of the intensities in the dimer and monomer bands, minus the fraction of the dimeric Prx2 in the lane with no H_2_O_2_ (which reflects the amount of lysis-induced oxidation in the sample). Bar graph plus dot plot shown in Supplementary Fig. 23. Data points represent mean value ± S.E.M for three biological replicates. ns: not significant at *p* < 0.02; two-tailed student’s *t*-test

To examine whether cytosolic expression of the probe perturbs the native, H_2_O_2_-clearing activity of endogenous Prx2, we stimulated cells with and without the fluorescent probe with various amounts of external H_2_O_2_, subjected the lysates from these cells to non-reducing, denaturing gel electrophoresis (i.e., non-reducing SDS-PAGE) followed by immunoblots for Prx2, and quantified the fraction of dimerized Prx2 in each sample (Fig. 3a–c). These membranes were also blotted for hyperoxidized Prx in order to ensure that no hyperoxidized species formed during these experiments (Supplementary Fig. 1,2). In lysates from these cells, two clear protein bands appeared that corresponded to reduced Prx2 monomers (21 kDa) and oxidized dimers (42 kDa) in the cytosol. Comparison of Fig. 3a with 3b indicates that expression of the probe does not shift the onset of native Prx2 dimerization.

More detailed examination of these redox western blots, provided in full as Supplementary Fig. 1,3, revealed several higher-molecular weight protein bands that likely correspond to the reduced probe monomers (75 kDa), oxidized heterodimers between probe and Prx2 monomers (100 kDa) and oxidized homodimers between two identical probe molecules (150 kDa). In order to observe the native state of the probe, lysates from cells expressing the probe were subjected to blue-native gel electrophoresis (BN-PAGE) and immunoblotted for both Prx2 and green fluorescent protein (Supplementary Fig. 1,4). No bands greater than 150 kDa were detected in the native blots, which suggests that the probe molecules do not assemble into high-molecular weight oligomers in these cells under the conditions used in our experiments.

### Probe responds to low levels of intracellular H_2_O_2_

**Fig. 4:**
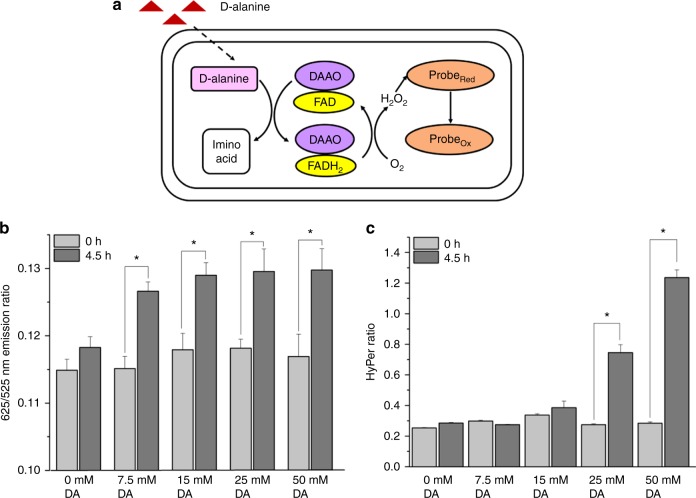
Sensitivity of Prx2-based probe compared to HyPer in response to D-amino acid oxidase (DAAO)-generated cytosolic H_2_O_2_. **a** Schematic representation of production of cytosolic H_2_O_2_ from DAAO. D-alanine (DA) is converted to an imino acid via reaction with DAAO; H_2_O_2_ is produced as a by-product of this reaction **b** 625/525 nm emission ratio of Prx2-based probe in response to various D-alanine concentrations. **c** HyPer ratio (defined here as the ratio of emission at 525 nm upon excitation at 488 nm divided by the emission at 525 nm upon excitation at 415 nm) in response to the same D-alanine concentrations. Bar graphs plus dot plots shown in Supplementary Fig. 23. Data points represent mean emission ratio ± S.E.M. for 18 cells and reflect results from two biological replicates. *p < 0.02; two-tailed student’s *t*-test

To progress beyond convenient, yet unrealistic, bolus additions of H_2_O_2_ we used the enzyme D-amino acid oxidase (DAAO), which has been previously used to produce continuous, tunable amounts of H_2_O_2_ within mammalian cells^35–37^. We expressed this enzyme in HeLa cells that contained either the Prx2-based probe or HyPer, a genetically-encoded H_2_O_2_ probed based upon the bacterial transcription factor OxyR, which is known to react with H_2_O_2_ with a second order rate constant nearly two orders of magnitude slower than Prx2^38^. Upon expression of DAAO in cell lines with the fluorescent probes, we supplied cells with various amounts of D-alanine, a substrate of DAAO, which resulted in steady production of H_2_O_2_ over a 4.5-h time period (Fig. 4a and Supplementary Fig. 1,5). We then measured the fluorescent signal from the two probes in each cell line over the same time period, and compared the initial signal with the final signal for each concentration of D-alanine (Fig. 4b, c and Supplementary Fig. 1,6). In these experiments, the signal from the Prx2-based probe increased significantly compared to baseline value even at the lowest concentration of D-alanine concentrations, whereas the HyPer signal did not increase significantly compared to the baseline value at D-alanine concentrations below 25 mM. In addition to this result, we observed that the signal from the Prx2-based probe appeared to saturate at D-alanine concentrations above 15 mM, whereas the HyPer signal gradually increased as the D-alanine concentration increased. Together, these results suggest that the Prx2-based probe responds to lower intracellular amounts of H_2_O_2_ compared to HyPer.

### Probe responds to decreases in TrxR activity

**Fig. 5:**
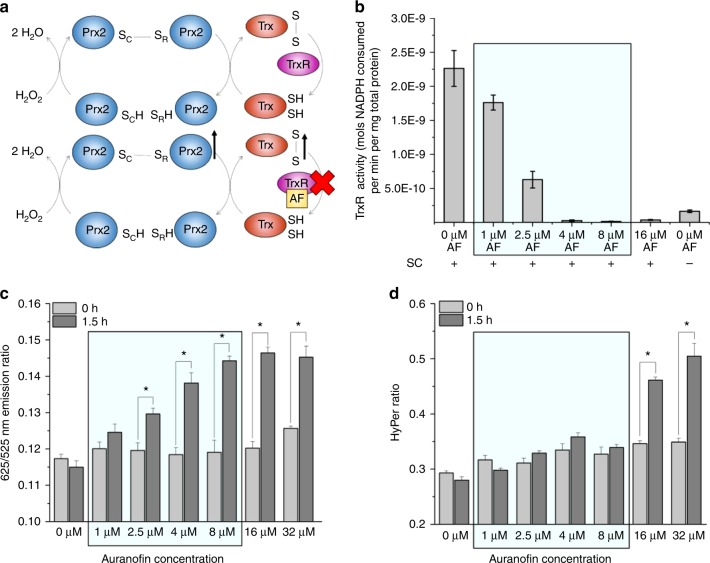
Effect of thioredoxin reductase inhibitor auranofin (AF) on response of fluorescent probe. **a** Schematic representation of effect of auranofin on human Prx2 oxidation status. As auranofin inhibits thioredoxin reductase, oxidized thioredoxin accumulates in the cell, which prevents reduction of oxidized Prx2 and causes continuous accumulation of oxidized Prx2 over time. **b** Activity of human thioredoxin reductase from HeLa cells treated with various concentrations of auranofin (**c**) 625/525 nm emission ratio of HeLa cells with Prx2-based probe upon incubation of different concentrations of auranofin for 90 min. Data points represent mean value ± S.E.M. for two biological replicates with two technical replicates each. **d** HyPer ratio of HeLa cells with HyPer probe upon incubation of different concentrations of auranofin for 90 min. Blue shaded region in plots represents range of auranofin concentrations at which significant inhibition of TrxR was observed. Bar graphs plus dot plots shown in Supplementary Fig. 23. Data points represent mean emission ratio ± S.E.M. for 18 cells and reflect results from two biological replicates. *p < 0.02; two-tailed student’s *t*-test

We next sought to determine the sensitivity of the Prx2-based probe to changes in Trx/TrxR activity. Since endogenous Prx2 utilizes the Trx/TrxR system for reduction of oxidized protein, we hypothesized that the Trx/TrxR system would also reduce probe molecules, and that inhibition of this reduction machinery would result in rapid accumulation of oxidized probe molecules coupled with an increased emission ratio from the probe. In order to test this hypothesis, we treated cells expressing either the Prx2-based probe or HyPer with the known TrxR inhibitor and cancer therapeutic compound auranofin^8,11^, which has been previously shown to cause the accumulation of oxidized Prx2 dimers in human cells^21^ (Fig. 5a). Since HyPer primarily utilizes glutaredoxin for reduction of oxidized protein^39^, we expected that this probe would be less sensitive to auranofin induced perturbations, and would only respond after significant accumulation of oxidized Prx2, which would allow HyPer to compete more effectively for intracellular H_2_O_2_. As expected, when HeLa cells were treated with auranofin, the activity of TrxR in the lysate decreased in a dose-dependent manner (Fig. 5b and Supplementary Fig. 1,8). Concomitant with this decrease in TrxR activity, the signal from the Prx2-based probe increased significantly compared to the baseline value at inhibitor concentrations above 1 μM, and quickly saturated at higher concentrations (Fig. 5c). In contrast, the HyPer signal only increased significantly from the baseline value at concentrations at or above 16 μM, and continued to increase as the inhibitor concentration increased (Fig. 5d). This change also corresponded to an increase in the intensity of Prx2 dimers as well as probe-based dimers, which started to accumulate on western blots at inhibitor concentrations above 8 μM (Supplementary Fig. 1,9). Together, these observations suggest that the Prx2-based probe rapidly responds to changes in the activity of the Trx/TrxR system, and support the hypothesis that HyPer, which primarily utilizes glutaredoxin for reduction, only responds significantly to the treatment after accumulation of oxidized Prx2 occurs.

### Probe responds to therapeutic-induced H_2_O_2_ elevation

**Fig. 6:**
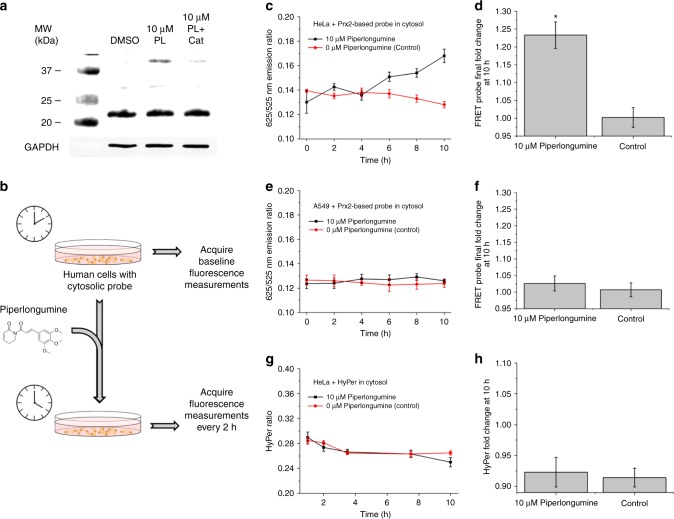
Response of Prx2-based probe and HyPer to the redox therapeutic compound piperlongumine (PL). **a** Human Prx2 in monomeric and dimeric form upon exposure to 10 μM piperlongumine, solubilized using dimethyl sulfoxide (DMSO), or an equivalent volume of DMSO without the drug. Endogenous glyceraldehyde 3-phosphate dehydrogenase (GAPDH) was simultaneously analyzed in these blots to assess the uniformity of lysate loading and protein content. **b** Schematic representation of piperlongumine experiment. **c** 625/525 nm emission ratio as a function of time in HeLa cells expressing the FRET probe upon incubation with either 10 μM piperlongumine in DMSO (black line), or an equivalent volume of DMSO (red line) over the course of 10 h in a total volume of 2.5 mL. **d** Emission ratio fold change at 10 h for the two different conditions. **e** 625/525 nm emission ratio as a function of time in A549 cells expressing the FRET probe upon incubation with either 10 μM piperlongumine in DMSO (black line), or an equivalent volume of DMSO (red line) over the course of 10 h in a total volume of 2.5 mL. **f** Emission ratio fold change at 10 h for the two different conditions. **g** HyPer ratio as a function of time from HeLa cells expressing HyPer upon incubation with either 10 μM piperlongumine in DMSO (black line), or an equivalent volume of DMSO (red line) over the course of 10 h in a total volume of 2.5 mL. Data points represent mean ratio ± S.E.M. for nine cells and reflect three biological replicates. **h** HyPer ratio fold change at 10 h for the two different conditions. Data points for time course experiments represent mean ratio ± S.E.M. for nine cells and reflect three biological replicates. Data points for fold change plots represent mean fold change ± S.E.M. for 27 cells and reflect three biological replicates. Bar graphs plus dot plots shown in Supplementary Fig. 23. *p < 0.02; two-tailed student’s *t*-test

In our last set of experiments, we asked if we could use the Prx2-based probe to detect redox perturbations associated with the redox therapeutic piperlongumine^13^. Recently, Huang et al.^14^. demonstrated that treatment of human epithelial cells with 10 μM piperlongumine results in the elevation of oxidized Prx2 dimers in addition to irreversible S-glutathionylation of proteins and cell death^14^. The authors of this study also showed that upon over-expression of catalase in the cytosol of these cells, piperlongumine treatment resulted in significantly less oxidized Prx2 in addition to lower cytotoxicity, which suggests that elevation of cytosolic H_2_O_2_ is central to the toxicity of piperlongumine. In order to assay for increased intracellular H_2_O_2_ after the addition of piperlongumine, we incubated a culture of HeLa cells with the drug for a period of 10 h and immuno-blotted the lysates for Prx2. Treatment of these cells with a dose of piperlongumine that was shown in past work^13^ to selectively kill several kinds of cancer cells caused an observable increase in the level of Prx2 dimerization compared to the control case (Fig. 6a). We next stably expressed the Prx2-based probe in the cytosol of HeLa cells and recorded the emission ratio of the sensor in the cells at various points in time after addition of piperlongumine (Fig. 6b). Upon addition of the drug to the cell culture media, the emission ratio of the probe began to increase after approximately 4–6 h, and steadily increased for the remainder of the 10-h incubation period (Fig. 6c, d). This signal was also reversible upon introduction of additional catalase in the cytosol (Supplementary Fig. 20). We then stably expressed the Prx2-based probe in the cytosol of A549 cells, which are known to overexpress certain antioxidant proteins as a result of hyperactive nuclear factor erythroid 2-related factor 2 (Nrf2)^40,41^, and repeated the previous experiment. As expected, upon stimulation with the drug, the signal from the probe did not increase, suggesting a lower cytosolic H_2_O_2_ concentration in response to the drug (Fig. 6e, f). In our last set of experiments, we stably expressed the H_2_O_2_ probe HyPer in the cytoplasm of HeLa cells and performed the piperlongumine experiment one more time. Upon stimulation with the drug, the HyPer emission ratio did not significantly change from the control case over the 10-h incubation period (Fig. 6g, h). These results agree with the results obtained by Huang et al.^14^, and suggest that the cytoplasmic H_2_O_2_ flux required for this Prx2 oxidation is below the detection limit of HyPer.

## Discussion

Due to the variety of roles that different oxidants play in both healthy and diseased cells, there is significant interest in the development of new fluorescent probes that can isolate the effects of therapeutic agents that perturb these species. Synthetic small molecule fluorescent probes for H_2_O_2_ have been developed, but many of these probes lack specificity and/or sensitivity for H_2_O_2_^42–45^. Similarly, several protein-based fluorescent probes that utilize the H_2_O_2_-sensitive protein domains of OxyR and Orp1 have been reported and have been successfully applied to study the effects of several H_2_O_2_-mediated processes in human cells^38,39^. Because Prxs react with H_2_O_2_ much more rapidly than these probes, some processes that only slightly elevate H_2_O_2_ may require a more highly reactive probe^38^. Recently, Morgan et al.^22^. designed a fluorescent probe based upon the redox relay between the yeast peroxiredoxin Tsa2 and the fluorophore roGFP2 and applied it in yeast, where it was more sensitive than other probes based on less reactive peroxidase domains^22^. In this study, we designed a probe based on human Prx2, the dominant peroxiredoxin species in human cells; this design allowed us to accurately track small increases in intracellular H_2_O_2_ that could not be detected using state-of-the art genetically-encoded probes.

Based on our choice of Prx2 as the reactive domain of the probe, we expected that the fluorescent probe would react with a similar specificity towards H_2_O_2_ and in a similar responsive range as the endogenous protein. Indeed, our experiments with the purified probe protein suggest that the probe has very high selectivity for H_2_O_2_ over other oxidants, with the exception of TBHP, which also oxidizes endogenous Prxs. In agreement with previous measurements from peroxiredoxin-based probes, TBHP caused an increase in the signal from our probe, but the other oxidants tested did not. The emerging literature related to ferroptosis^46,47^ has drawn new attention to the intersection of iron homeostasis and dysregulation with intracellular levels of various peroxides. Use of the Prx probe we report to study alterations in iron homeostasis will warrant additional validation experiments to understand whether and how such differences influence the signal from the probe.

Our experiments with different pH buffers suggest that the Prx FRET probe has better pH stability than HyPer, as a result of the choice of fluorophores rather than the choice of H_2_O_2_ reactive domains. As originally reported, HyPer’s excitation ratio (500 nm/420 nm) in the reduced form at pH 7.4 is greater than its excitation ratio in the fully oxidized form at pH 7.2, and pH controls are always necessary. Supplementary Fig. 7 shows that the Prx probe’s emission ratios in the reduced form are always less than the emission ratios exhibited by the oxidized form. The magnitude of the error bars in Supplementary Fig. 7 demonstrate that pH controls should only be necessary in the subset of experiments that show small FRET increases comparable with variability in the values for the reduced probe as a function of pH.

Our measurements from the cytosolic probe upon stimulation with different amounts of H_2_O_2_ indicate that the fluorescent Prx2-based probe has a responsive range similar to that of the native, unmodified protein. Reactivity with H_2_O_2_ does not appear to be altered by fusion with two fluorescent protein domains. Moreover, the relatively low cell-to-cell variability of the probe’s signal (approximately 5% of the maximum fold change in these experiments) allowed us to visualize very small differences in external H_2_O_2_. Based on bolus addition experiments, the probe reached saturation upon exposure to approximately 30 fmol H_2_O_2_ per cell. Given that peroxiredoxin does not become hyperoxidized^21^ at this concentration (Supplementary Fig. 1,2) the gradient of H_2_O_2_ across the plasma membrane of HeLa cells is approximately 650-fold^48^, and this extracellular concentration corresponds to an intracellular concentration of approximately 15 nM. If the H_2_O_2_ perturbation in a process of interest is extreme enough that significant hyperoxidation or saturation of oxidized Prx2 would result, an ultrasensitive probe such as the one we report here would not be needed, and other H_2_O_2_ probes, such as HyPer or roGFP2-Orp1, may serve as the better choice. We advocate experiments using complementary techniques such as immunoblots with antibodies specific to hyperoxidized Prx in cases where extreme intracellular levels of H_2_O_2_ are suspected, or to rule these out.

Similar to other genetically-encoded fluorescent probes for H_2_O_2_, we observed a slight shift in the fluorescence ratio of the probe before addition of any oxidant in experiments occurring on the minutes-timescale where samples were imaged frequently. In Fig. 2 and in Supplementary Figures 9 and 1,0, a slight upward drift of the emission ratio is apparent. Examination of the data used to calculate emission ratios shows that the upward drift is a true FRET signal rather than a ratio change attributable to photobleaching. This monotonic upward drift was not observed in experiments that occurred on the hours-timescale with less frequent imaging (Fig. 6, Supplementary Figure 1,6). In order to aid in interpretation of data in all of these experiments, we recommend the use of control experiments in which media without any stimulant is added to cells so that changes in the emission ratio of the cells after addition of the stimulant can be compared with the baseline signal over the same period of time.

Given that the concentration of endogenous Prxs is on the order of 1 × 10^−4^ M^16,18^, and that the typical concentration of fluorescent protein constructs is on the order of 1 × 10^−6^ M^49^, we hypothesized that the presence of the probe would not significantly affect the level of oxidized Prx2 in the cytosol upon stimulation with H_2_O_2_. As expected, the concentration of the intracellular probe was determined to be approximately 1 × 10^−6^ M (Supplementary Fig. 21), and expression of the probe in the cytosol did not significantly affect the observed amount of oxidized endogenous Prx2 dimers upon treatment with H_2_O_2_, although we anticipate that much higher expression levels of the probe may impact the activity of the endogenous protein. Currently, it is not known whether recombinant peroxiredoxin constructs assemble into high-molecular weight oligomers upon expression in mammalian cells^50^ in a similar fashion to analogous probes in yeast^22^. While we did see evidence of high molecular weight oligomers of endogenous Prx2 in our native gel electrophoresis experiments in samples probed with an antibody specific to human Prx2 (in agreement with previous studies conducted with lysates from mammalian cells^51,52^), we did not see any significant formation of high molecular weight oligomers in samples probed with antibody against GFP, which suggests that the probe does not form these oligomeric complexes under the conditions we used and does not require them to function in these cells.

The probe developed in this work was also characterized in terms of sensitivity towards intracellular H_2_O_2_ generation. Most current genetically-encoded H_2_O_2_ sensors (e.g., HyPer and roGFP2-Orp1) are based on protein domains that react with H_2_O_2_ with a relatively low rate coefficient (on the order of 1 × 10^5^ M^−1^s^−1^)^38^. In contrast, Prx2 reacts with H_2_O_2_ with a rate coefficient nearly two orders of magnitude higher (on the order of 1 × 10^7^ M^−1^s^−1^)^17^. In combination with the abundance of Prx2 in the cytosol of human cells (on the order of 1 × 10^−4^M)^16,18^ and reduction by Trx, Prx2 kinetically outcompetes other potential reaction partners for H_2_O_2_ in the cell, which allows it to respond to very small fluctuations in the H_2_O_2_ level before other proteins that contain less reactive cysteines can react with H_2_O_2_. In our experiments with the cytosolic H_2_O_2_ generator DAAO, cells expressing the Prx2-based probe responded to lower amounts of the D-alanine compared to cells expressing HyPer over the timescale of hours (Supplementary Fig. 1,6) as well as immediately after D-alanine addition (Supplementary Fig. 1,7). We observed that higher concentrations of D-alanine were required to induce changes in HyPer’s ratiometric signal in comparison with a previous study^37^. This difference may result from differences in the expression levels of DAAO or HyPer or both; each are expected to impact the results^37,49^. In order to control for variability expected as a function of DAAO’s expression level, we transfected cell lines with equal amounts of DAAO in order to compare the two fluorescent probes. Our results from this experiment suggest that Prx2-based probe responds to lower levels of intracellular H_2_O_2_ than HyPer, and based on the results obtained from Morgan et al.^22^., we expect that this probe would also out-compete other genetically-encoded probes for H_2_O_2_ with similar reactivity as HyPer.

After detailed characterization of the probe’s reactivity with H_2_O_2_, we used it to detect perturbations caused by redox cancer therapeutics. Recently, several studies have suggested that Prxs may serve as the focal point for many redox-based stress response pathways due to their ability to not only rapidly clear H_2_O_2_ from the cytosol, but also to directly affect the activity of other redox-regulated proteins in the cytosol through disulfide exchange mechanisms^53,54^. Cells utilize reductase enzymes such as Trx and TrxR to maintain a large pool of reduced Prxs in the cytoplasm which can respond to small fluctuations in H_2_O_2_. One way redox cancer therapeutics are thought to perturb this Prx/Trx/TrxR pathway in cancer cells is through inhibition of the reduction pathways that cells used to regenerate reduced antioxidant enzymes, which results in accumulation of oxidized peroxiredoxin species that can participate in signaling reactions^9,11^. In our experiments with the TrxR inhibitor auranofin, we treated cancer cells with an amount of inhibitor sufficient to cause near-complete inhibition of TrxR activity and observed a significant increase in the emission ratio of our probe. Other redox therapeutics also cause an increase in the intracellular level of H_2_O_2_ that results in selective toxicity to a wide variety of tumors^12^. In our experiments with the compound piperlongumine, we treated cancer cells with an amount of drug previously shown to induce elevation of cytosolic H_2_O_2_ that was essential to toxicity over a 48 h period^14^ and observed a significant increase the emission ratio of our probe, which was reversible upon over-expression of catalase in the cytosol. In both of the above examples, we were unable to see any change in the emission ratio from HyPer, which suggests that the probe developed in this work has the capability to sense these slight, but physiologically-relevant, redox therapeutic-induced perturbations.

In conclusion, we created and characterized an ultrasensitive genetically-encoded probe for H_2_O_2_ based on human Prx2. We used this probe to track changes in TrxR activity as well as intracellular H_2_O_2_ generation in response to the cancer therapeutics auranofin and piperlongumine, respectively. We also compared the signal of this probe to that of another commonly used probe in the field, which was unable to detect the slight perturbations associated with these treatments. We believe that these capabilities highlight the potential of this probe as a valuable tool for the measurement of H_2_O_2_-mediated processes in human cells, and uniquely position it to offer insight into the mechanism of action a number of different therapeutic compounds in which elevation of oxidative species is thought to play a role^8,9,11,55^.

## Methods

### Reagents

All reagents were obtained from Sigma-Aldrich unless otherwise specified.

### Construction of fluorescent probe

The cloning sequences for Clover, mRuby2, and Prx2 were isolated from their original plasmids via polymerase chain reaction (PCR). pcDNA3.1-Clover-mRuby2 was a gift from Kurt Beam (Addgene plasmid # 49089); pQTEV- PRDX2 was a gift from Konrad Buessow (Addgene plasmid # 31338). Next, splice overlap extension PCR reactions were used to add 30-40 nucleotide sequences to the 3’ of the Clover gene and the 5’ end of the mRuby2 gene. These oligonucleotides contained the sequences for the linker sequences between the fluorescent protein and Prx2 domain and also an overlap region between the fluorophore and the Prx2 domain for the Gibson assembly reaction. Next, a second round of splice overlap extension PCR reactions were used to add vector overlap sequences to the 5’ end of the Clover gene and the 3’ end of the mRuby2 gene. In order to create the final fusion product, the three gene fragments were assembled into a linearized vector via Gibson assembly. In order to obtain the linearized vector for the Gibson assembly reaction, the pcDNA3.1(+) mammalian expression vector (ThermoFisher Scientific) was digested with EcoRI/XhoI (New England Biolabs) for 15 min at 37 °C. The three gene fragments were then co-incubated with the linearized vector and Gibson assembly master mix (New England Biolabs) at 60 °C for 60 min.

### Cell culture and stable cytosolic expression of probe

Hela cells (ATCC^®^ CCL-2) and HEK-293 cells (ATCC^®^ CRL-1573) lines were cultured in Dulbecco’s Modified Essential Medium (DMEM; Lonza) with 10% Fetal Bovine Serum (FBS; ATCC) at 37 °C, 95% air and 5% CO_2_. Cell lines obtained directly from ATCC were not authenticated or tested for mycoplasma. Media in the culture dish was replaced with fresh media every 2–3 days, and the cells were passaged every 4–5 days. In order to create a cell line with stable expression of the fusion construct, the fluorescent protein was constitutively expressed under control of the human cytomegalovirus (CMV) promoter with the pLJM1 lentiviral vector; the original plasmid, pLJM1-EGFP, was a gift from David Sabatini (Addgene plasmid # 19319). A HEK 293FT cell culture was first transfected with the plasmid of interest along with lipofectamine 2000 (Invitrogen) in Opti-MEM media (ThermoFisher Scientific) via the manufacturer’s instructions. 24 h after transfection, the media in the HEK culture was replaced with DMEM supplemented with 10% FBS; after an additional 24 h of incubation, the media in the HEK culture was collected and the supernatant containing the lentiviral particles was isolated. The lentiviral particles were added to a separate HeLa cell culture along with 6 μg/mL polybrene and the cells were incubated for approximately 72 h. The HeLa cell were then removed from the dish with 0.25% Trypsin-EDTA (ThermoFisher Scientific) and re-plated onto a 10 cm dish with 8 mL of DMEM supplemented with 10% FBS and 6 μg mL^−1^ puromycin. Cells were grown in this selective media for a period of 4-5 days before the cell stocks were prepared.

### Characterization of probe reactivity in human cells

Prior to the experiment, either 1.75 × 10^5^ cells or 3.5 × 10^5^ cells were seeded into a single well of a 6-well plate (Corning) with approximately 9.5 cm^2^ of available growth area. After either 24 h or 48 h of incubation, respectively, cells were transiently transfected with 1.5 μg of the plasmid of interest with Lipofectamine 2000 according to the manufacturer’s instructions. After 24 h of incubation, cells were washed once with 1 mL of phosphate buffered saline (PBS) (1×) and then placed in 1 mL of Roswell Park Memorial Institute (RPMI) 1640 media with no phenol red (ThermoFisher Scientific). For each data set, baseline signal was acquired for 2.5 min; once 2.5 min had elapsed, 1 mL of RPMI 1640 medium with 40 μM H_2_O_2_ was added to the cells in each well to reach a final concentration of 20 μM H_2_O_2_ in a total volume of 2 mL a final cell density of 3.5 × 10^8^ cells L^−1^. Approximate values for mols H_2_O_2_ per cell were calculated by dividing the concentration of H_2_O_2_ (in units of mols L^−1^) by the cell density (expressed in cells L^−1^).

### Characterization of cytosolic probe dose response to H_2_O_2_

Prior to the experiment, either 1.75 × 10^5^ cells or 3.5 × 10^5^ cells stably expressing the fluorescent Prx2 probe were seeded onto each well of a 6-well plate with approximately 9.5 cm^2^ of available growth area. The day of the experiment, cells were washed with 1 mL of PBS (1×) and then placed in 1 mL of RPMI 1640 media with no phenol red. For each data set, baseline signal was acquired for 2.5 min. After 2.5 min had elapsed, 1 mL of RPMI 1640 medium with an H_2_O_2_ concentration of 40, 20, 10, 2, or 0 μM H_2_O_2_ was added to the cell culture to reach final H_2_O_2_ concentrations of 20, 10, 5, 1, and 0 μM H_2_O_2_, in a total volume of 2 mL along with a final cell density of 3.5 × 10^8^ cells L^−1^. Fluorescent images were acquired in the same manner as described as above. As before, approximate values for the mol H_2_O_2_ per cell were calculated by dividing the concentration of H_2_O_2_ (in units of mol L^−1^) by the cell density (expressed in cells L^−1^).

### Production of intracellular H_2_O_2_ from D-amino acid oxidase (DAAO)

In order to continuously produce H_2_O_2_ in the cytosol of HeLa cells, DAAO, which we previously^14^ cloned into the pLJM1-EGFP construct (Addgene plasmid # 19319), was stably expressed under control of the CMV promoter in the cytosol of HeLa cells containing either the Prx2-based probe or HyPer with the procedure described above. Cells were grown in the selective media for a period of 2 days before the cells were passaged again. The day before the experiment, 3.5 × 10^5^ cells were seeded onto the surface of a 6-well plate. The day of the experiment, cells were washed once with PBS (1×) and supplemented with 1 mL of RPMI 1640 (1×) containing 5 μM flavin adenine dinucleotide (FAD). Before addition of D-alanine (the DAAO substrate) to the wells, images in each fluorescent channel were acquired for each well. After these images were acquired, D-alanine (dissolved in RPMI 1640) was added to the wells to reach final D-alanine concentrations of 50, 25, 15, and 7.5 mM in a total volume 2 mL; as a control, an equivalent volume of RPMI 1640 with no D-alanine was added to a separate cell sample. Images were acquired in each fluorescent channel in each well after 1, 2, and 4.5 h.

### Treatment with auranofin and piperlongumine

For experiments that involved the TrxR inhibitor auranofin (Cayman Chemical Company), solid auranofin was dissolved in sterile-filtered DMSO to make a 32 mM stock solution the day of the experiment. The auranofin stock solution was then added to culture medium comprised of DMEM supplemented with 10% FBS to reach final auranofin concentrations of 32, 16, 8, 4, and 1 μM in a total volume of 2 mL culture medium. Pure dimethyl sulfoxide (DMSO) was used as a control. Cells were then incubated at 37 °C for a period of 90 min. After incubation, the signal in each fluorescent channel was acquired in three different regions of each well. For experiments that involved the cancer therapeutic piperlongumine, solid piperlongumine (Cayman Chemical Company) was dissolved in DMSO to make 30 mM stock solution the day of the experiment. Before addition of the piperlongumine to the cell culture, images in each fluorescent channel were acquired for each well. After these images were acquired, the piperlongumine stock solution was then added to the media to reach a final piperlongumine concentration of 10 μM in a total volume 2.5 mL; as a control, an equivalent volume of DMSO was added to a separate cell sample. Images were acquired in each fluorescent channel in each well every 2 h for a period of 10 h.

### Selenocystine assay for TrxR activity

Immediately after treatment with auranofin, cells were washed twice with PBS (1×) on ice then treated with 100 µL of 1% Triton x-100 supplemented with 1x HALT protease cocktail (ThermoFisher Scientific) to lyse the cells. The lysed cells were then re-pelleted at 12,000 g for 10 min. The supernatant of the solutions was then collected and the protein content in the solution was assessed via the bicinchoninic acid (BCA) assay. 40 μg of protein were then added to a solution containing 400 μM NADPH (VWR) and 800 μM selenocystine (ThermoFisher Scientific). The absorbance of the solution at 340 nm was then measured every 2 min for a period of 90 min.

### Gel electrophoresis and peroxiredoxin-2 western blots

For each experiment in which western blots were used, the adherent cells were first washed twice with PBS (1×). For experiments in which H_2_O_2_ was used, the liquid in each well was replaced with 1 mL of PBS and the H_2_O_2_ was added to the wells in the same manner as above. After approximately 30 s, the liquid in each well was aspirated and replaced with 2 mL of 100 mM methyl methanethiolsulfonate (MMTS) in order to block any free thiol groups as described previously. The plates were then incubated on ice for a period of 20-30 min, and washed twice with 1 mL PBS. Next, 100 µL of 1% Triton x-100 supplemented with 1x HALT protease cocktail was added to lyse the cells. The lysed cells were then re-pelleted at 12,000 g for 10 min. The supernatant of the solutions was then collected and the protein content in the solution was assessed via the BCA assay. 25 μg of protein was then loading into a tris-tricine acrylamide gel and subjected to SDS-PAGE under non-reducing conditions (i.e. with no β-mercaptoethanol in sample buffer). After the separated lysates were transferred to a PVDF membrane (Bio-Rad) (1 h at 100V), the blot was blocked and incubated with either goat primary antibody for Prx2 (R&D Systems, Catalog # AF3489) (at a dilution of 1:800) or goat primary antibody for GFP (R&D Systems, Catalog # AF4240) at 4 °C overnight and then with donkey anti-goat IR 688 (Licor) (at a dilution of 1:10,000) for 1 h at approximately 22 °C. The tagged proteins were then visualized on the Odyssey CLx Infrared Imaging System. GAPDH was used as a loading control for all blots. Uncropped version of all blots in main text shown in Supplementary Figure 22.

### Fluorescent measurements and image analysis

Adherent cells were imaged with an inverted Olympus IX81 widefield microscope with an Olympus plan fluorite 10x dry objective (NA = 0.30). A 200 W metal-arc lamp (Prior) was used as a power source. For ratiometric FRET measurements, two different filter sets were used: for Clover fluorescence measurements, a 488/6 nm excitation filter (Semrock) was used with a 525/40 nm emission filter (Semrock); for Clover-mRuby2 FRET fluorescence measurements, a 488/6 nm excitation filter was used with a 625/40 nm emission filter (Semrock). A 1000 ms exposure time at 25% lamp intensity was used to acquire images in both fluorescent channels. For image analysis, 16-bit.tif images were exported to ImageJ and regions-of-interest (ROIs) were then drawn around cells to be analyzed. In order to calculate the raw emission ratio in each ROI at each time point, the background-subtracted FRET fluorescence intensity was divided by the background-subtracted Clover fluorescence intensity in ImageJ.

For experiments in which the fluorescent probe HyPer was used, the following two filter sets were used: for short wavelength HyPer excitation, a 415/6 nm excitation filter (Semrock) with a 525/40 nm emission filter (Semrock); for long wavelength HyPer excitation, a 488/6 nm excitation filter with a 525/40 nm emission filter (Semrock). A 250 ms exposure time at 10% lamp intensity was used to acquire the images in both channels. ROIs were determined in images from both fluorescent channels as above. In order to calculate the ratiometric HyPer signal (i.e., the HyPer ratio) in each ROI at each time point, the background-subtracted fluorescence intensity from the 488 nm excitation channel was divided by the background-subtracted fluorescence intensity from the 415 nm excitation channel in ImageJ.

For all images, background-subtraction was performed in ImageJ with the rolling ball algorithm. In order to create the emission ratio image, the background-subtracted 16-bit.tif images for each fluorescent channel were imported into MATLAB and divided pixel-by-pixel.

### Statistical analysis

All experimental emission ratio values are reported as the mean value ± S.E.M. In order to calculate the mean raw emission ratio value for each sample, the raw emission ratio was calculated for the designated number of cells from the background-subtracted images for each timepoint; these values were then averaged to obtain the raw emission ratio values for these cells. Neither the brightest cells nor the dimmest cells were used for probe characterization. Probe fold change values are reported as mean value ± S.E.M.; mean probe fold change values were determined in a similar manner to the raw emission ratios. The normalized fold change values were determined from the average fold change values from the control and experimental samples. The error associated with these average ratios was propagated according to the following expression:

1 $${\sigma}_{\mathrm{NFC}} = ({\mathrm NFC}) \, {\ast} \, \sqrt{\left( \frac{{\sigma}_{\mathrm{FC}}}{\mathrm{FC}} \right)^{2} + \left( \frac{{\sigma}_{{{\mathrm FC}\left( {\mathrm control} \right)}}} {{{\mathrm FC}\left( {\mathrm control} \right)}} \right)^{2}}$$

where *σ*_NFC_, *σ*_FC_, and *σ*_FC(control)_ are the uncertainties in the normalized fold change, the peak fold change value, and the peak fold change value of the control sample (i.e., 0 μM H_2_O_2_), respectively. NFC, FC, and FC (control) are the normalized fold change value, the peak fold change value, and the peak fold change value of the control sample, respectively.

For the piperlongumine experiment, the final probe fold change values were determined from the average raw emission ratios at the final and initial time points. The error associated with these average ratios was propagated according to the following expression:

2 $$\sigma_{\mathrm{FC}} = \left( \mathrm{FC} \right) \ast \sqrt {\left( \frac{\sigma_{R\left( {t = 0} \right)}}{R\left( {t = 0} \right)} \right)^{2} + \left( \frac{\sigma _{R\left( {t = 10} \right)}}{R\left( {t = 10} \right)} \right)^{2}}$$

where *σ*_FC_, *σ*_*R*(*t*=0)_, and *σ*_*R*(*t*=10)_ are the uncertainties in the fold change, the raw emission ratio at 0 h, and the raw emission ratio at 10 h, respectively. FC, *R*(*t* = 0), and *R*(*t* = 10) are the fold change value, the raw emission ratio value at 0 h, and the raw emission ratio value at 10 h, respectively. In order to assess the significance of differences in the probe’s fold-change values in all experiments, a two-tailed student’s *t*-test was used at a significance level of *p* = 0.02.

### Data availability

The authors declare that the data supporting the findings of this study are available within the paper and its supplementary information files. All constructs created in this study are available upon request.

## Electronic supplementary material

Supplementary Information

Description of Additional Supplementary Files

Supplementary Data 1
