# Supplementary material for: Monitoring the action of redox-directed cancer therapeutics using a human peroxiredoxin-2-based probe
Source: Nat Commun. 2018 Aug 7;9:3145. doi: 10.1038/s41467-018-05557-y (PMC6081480; doi:10.1038/s41467-018-05557-y)
Supplement: Supplementary file 1 — Supplementary Information [file 41467_2018_5557_MOESM1_ESM.pdf]

Supplementary Information

Monitoring the action of redox-directed cancer therapeutics using a human peroxiredoxin-2-based probe

Langford et al.

Supplementary Figures

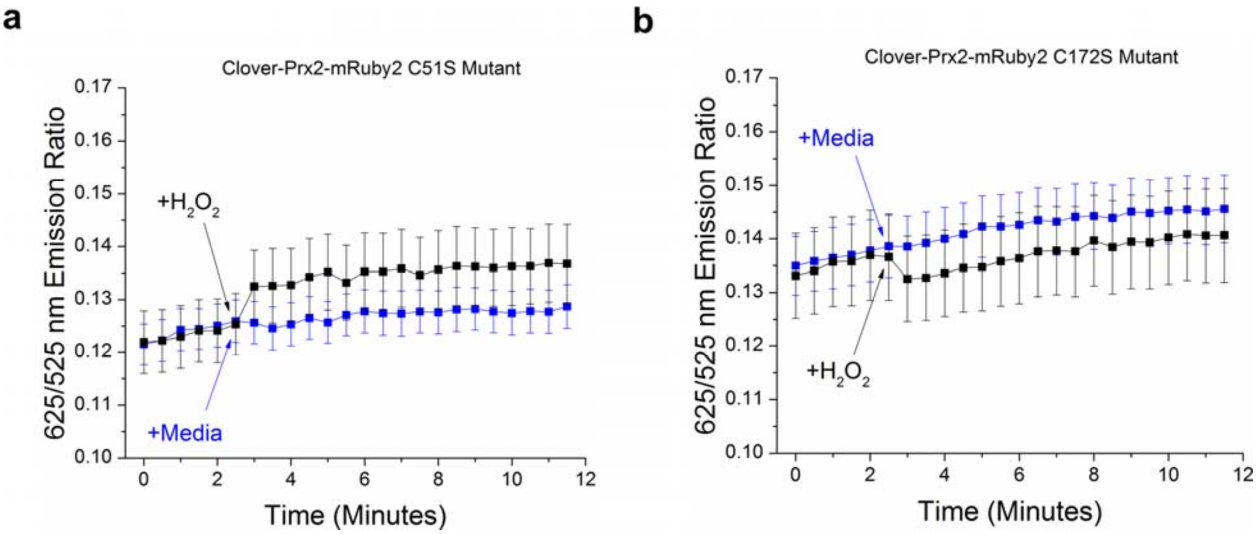

Supplementary Figure 1 | (**a**) 625 nm/525 nm emission ratio over time in HeLa cells with Prx2-based probe with the Prx2 resolving cysteine residue mutated to serine upon stimulation with a control bolus (i.e. just culture media) (blue line) or stimulation with approximately 60 x10^{-15} mol H_2O_2/cell (black line) in cells with the functional Prx2 probe. (**b**) 625 nm/525 nm emission ratio over time in HeLa cells with Prx2-based probe with the Prx2 catalytic cysteine residue mutated to serine upon stimulation with a control bolus (i.e. just culture media) (blue line) or stimulation with approximately 60 x10^{-15} mol H_2O_2/cell (black line) in cells with the functional Prx2 probe. Data points from all plots represent mean FRET ratio ± S.E.M. for 18 cells and are reflective of three biological replicates.

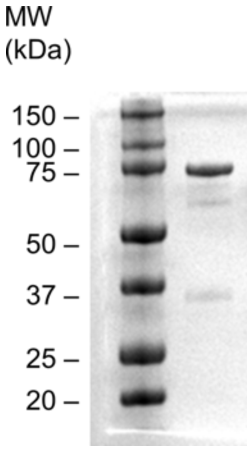

Supplementary Figure 2 | Purification of His-tagged Prx2-based probe from BL-21 bacterial cells. Cells were first expressed in BL-21 cells, lysed via sonication, and purified via an immobilized metal affinity column. Purified protein (2 μg) was then subjected to SDS-PAGE on a 9% acrylamide gel under reducing conditions and stained for protein. The single band at 75 kDa corresponds to the fluorescent probe protein.

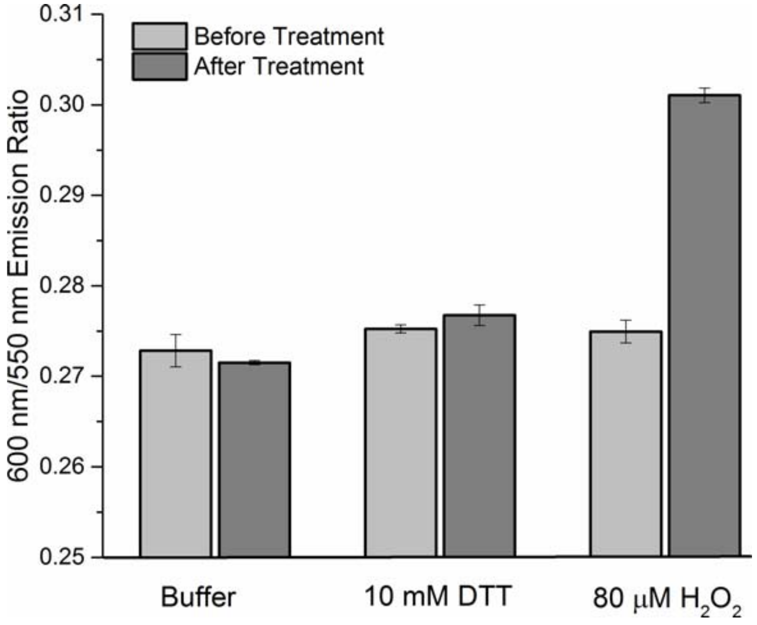

Supplementary Figure 3 | Characterization of purified Prx2-based probe fluorescence in response to DTT and H_2O_2. Purified probe (16 μM) was combined with either a bolus of reaction buffer solution (as a control), 10 mM DTT, or 80 μM H_2O_2. The 600 nm/550 nm emission ratio was measured immediately before and after each treatment. Error bars represents mean emission ratio ± S.E.M. for two independent experiments.

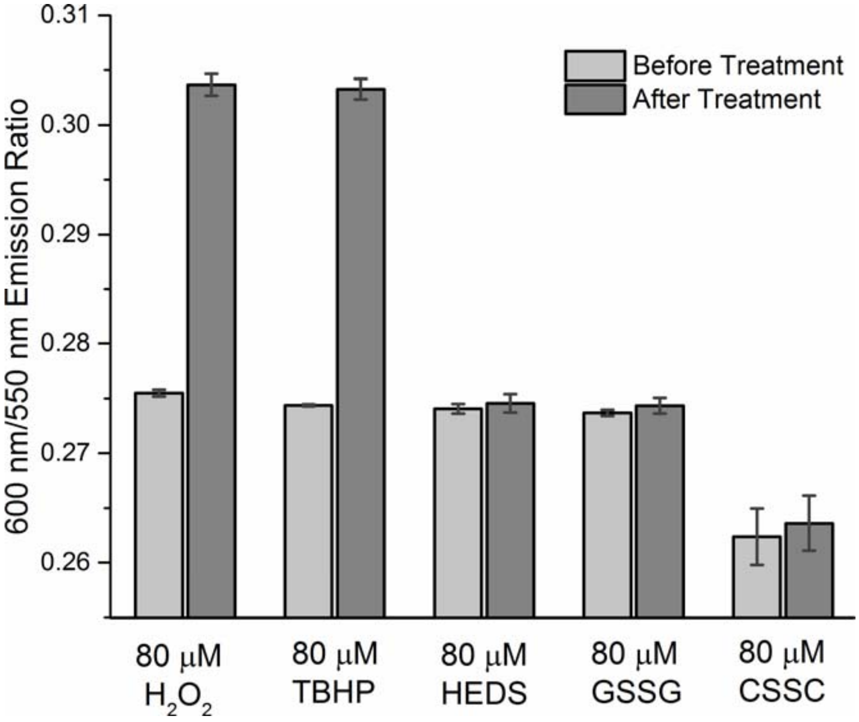

Supplementary Figure 4 | Characterization of purified Prx2-based probe fluorescence in response to different oxidants. Purified probe (16 μM) was combined with either 80 μM H_2O_2, 80 μM tert-butyl hydroperoxide (TBHP), 80 μM hydroxyethyl disulfide (HEDS), 80 μM oxidized glutathione (GSSG), or 80 μM oxidized cysteine (CSSC). The 600 nm/550 nm emission ratio was measured immediately before and after each treatment. Error bars represents mean emission ratio ± S.E.M. three independent experiments.

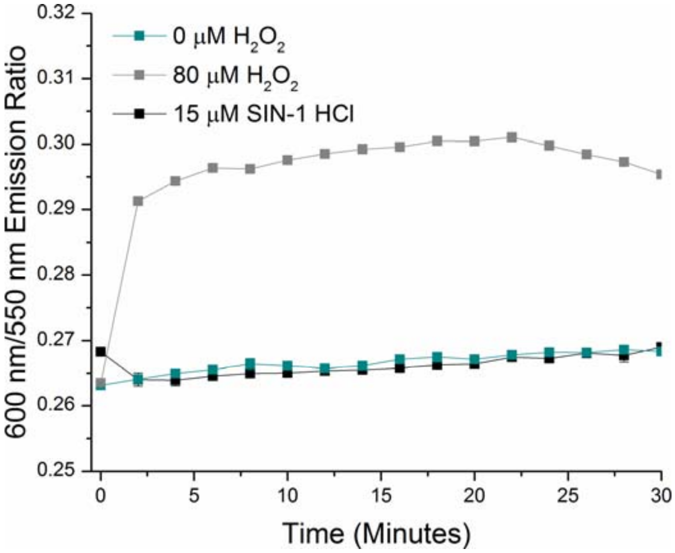

Supplementary Figure 5 | Characterization of purified Prx2-based probe fluorescence in response to H_2O_2 and peroxynitrite generation via 3-morpholinosydnonimine (SIN-1 HCl). Purified probe (16 μM) was treated with either H_2O_2 or SIN-1 HCl in a similar manner to that described by Morgan *et al.* The 600 nm/550 nm emission ratio was measured immediately before addition of the peroxynitrite generator and then again every 2 minutes for a total of 30 minutes after addition of the reagent. Error bars represents mean emission ratio ± S.E.M. three independent experiments.

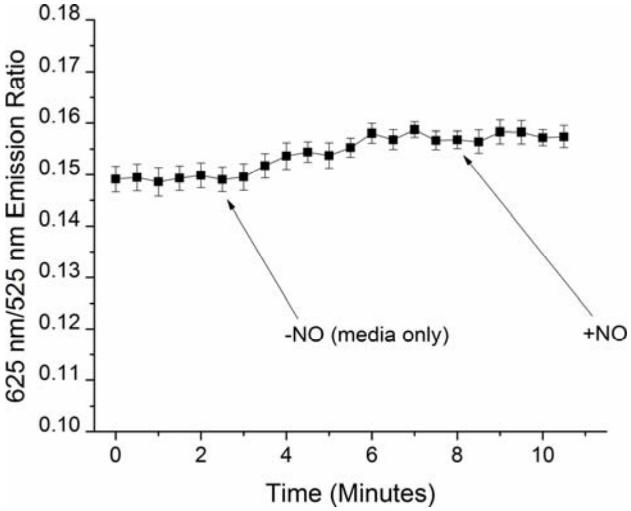

Supplementary Figure 6 | Fluorescent response of intact Hela cells with Prx2-based probe in response to external NO addition. 625 nm/525 nm emission ratio upon stimulation with approximately 1 μM NO. Approximately 2 x10^5 cells were seeded into each well (9.5 cm^2 of growth area) one day before the experiment. Cells were imaged the next day in RPMI 1640 media. Each well contained a total of 2 mL of solution after addition of the H_2O_2 solution. All data points represent the mean ratio ± S.E.M. for 9 different cells.

NO was generated using diethylamine NONOate (DEA/NO). In order to determine the concentration of nitric oxide in the DEA/NO 37°C phosphate buffered solution as a function of time, a kinetic model first utilized by Schmidt *et al.*[1] was used with kinetic parameters obtained from Kavdia *et al*[2]. This simple kinetic model takes into account the rate of formation of nitric oxide from DEA/NO at pH 37°C and pH 7.4 and the rate of autoxidation of nitric oxidation in solution when oxygen is present. The system of two differential equations that describe the rates of change of the concentrations of DEA/NO and nitric oxide were solved in MATLAB and the solution was used to estimate the concentration of nitric oxide in solution at each point in time. Based on this model, for an initial concentration of DEA/NO of 30μM, the concentration of nitric oxide reaches a peak value of approximately 1 μM after approximately 2.5 minutes

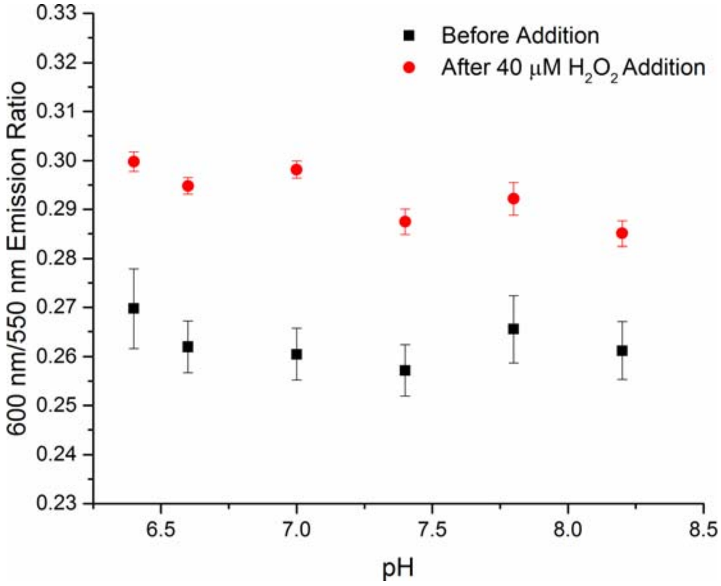

Supplementary Figure 7 | Characterization of purified Prx2-based probe fluorescence in response to buffers with different pH values. Emission ratio from purified probe (8 μM) was measured before and after 40 μM H_2O_2 bolus addition in buffers of 6 different pH values. Error bars represent mean emission ratio ± S.E.M. three independent experiments.

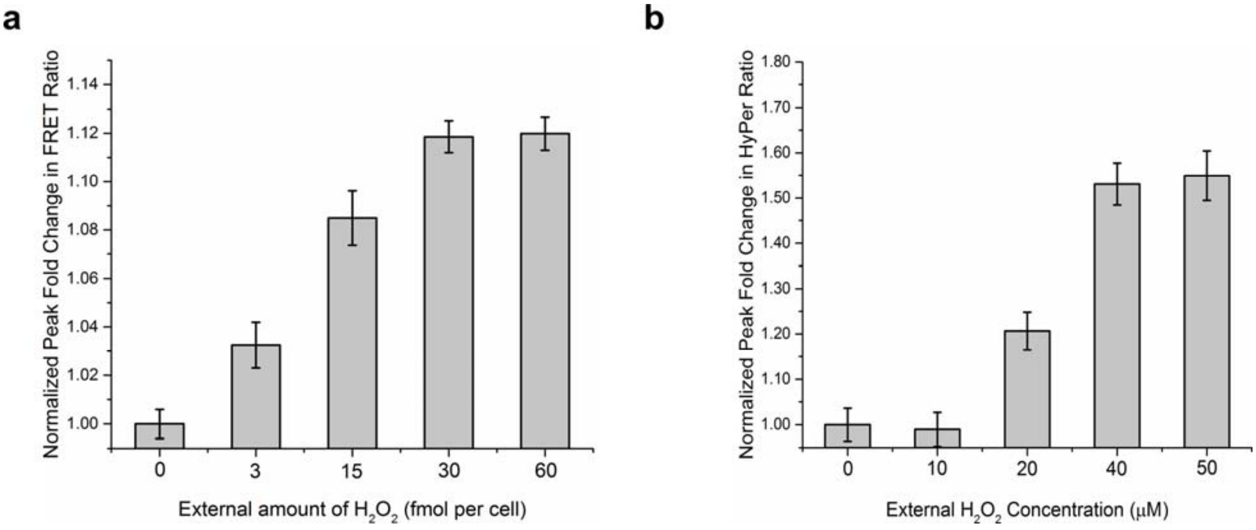

Supplementary Figure 8 | Normalized peak fold change of Prx2 based probe and HyPer in response various H_2O_2 bolus additions. (**a**) Normalized peak fold change of Prx2 based probe upon stimulation with different amounts of H_2O_2. Note that **Supplementary Figure 8a** is identical to **Figure 2f** in the main text and is shown here for comparison purposes with HyPer. (**b**) Normalized peak fold change of HyPer upon stimulation with various amounts of H_2O_2. Note that bolus additions below 10 μM H_2O_2 were not used since no response was observed from HyPer upon stimulation with 10 μM H_2O_2. Data points represent mean normalized peak fold change in HyPer ratio ± S.E.M. for 14 fields of views.

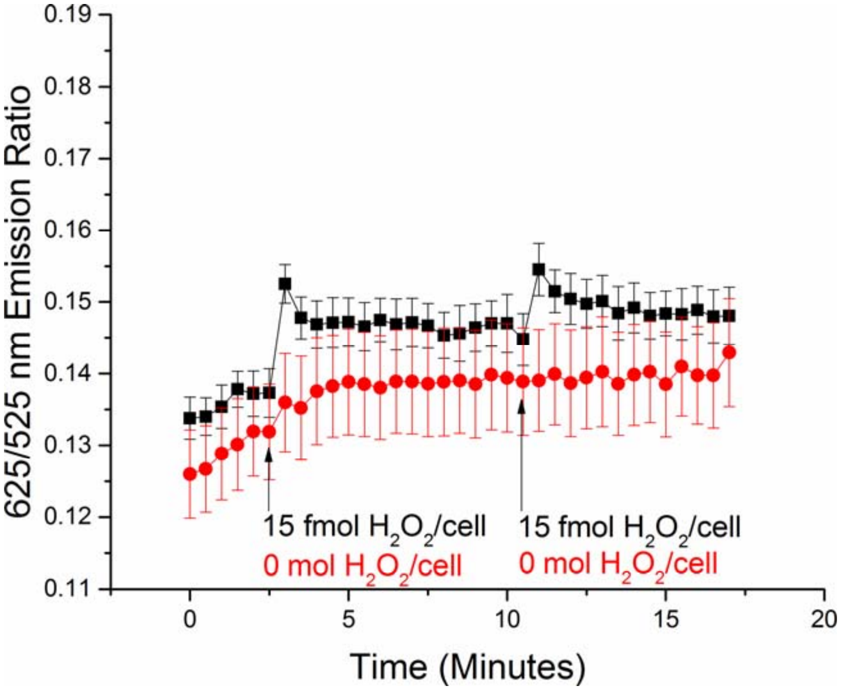

Supplementary Figure 9 | Fluorescent response of Prx2 probe after stimulation with subsequent bolus additions of H_2O_2. Approximately 2 x10^5 HeLa cells stably expressing the fluorescent Prx2 probe were seeded onto each well of a 6-well plate (9.5 cm^2 of growth area) two days before the experiment. Each well contained a total of 2 mL of solution after addition of the H_2O_2 solution. The day of the experiment, cells were imaged in RPMI media. Data points represent the mean ratio ± S.E.M. for 9 different cells for two biological replicates.

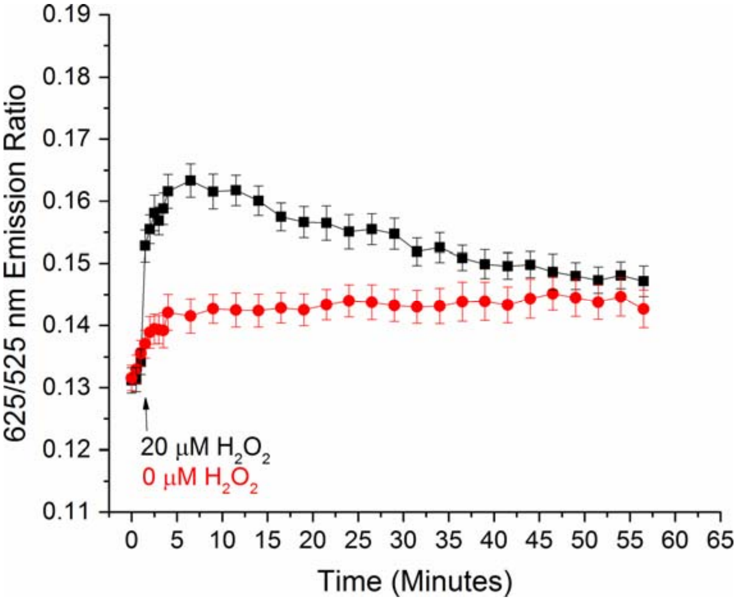

Supplementary Figure 10 | Fluorescent signal from Prx2-based probe over time upon exposure to a 20 μM H_2O_2 bolus. Approximately 2 x10^5 HeLa cells stably expressing the fluorescent Prx2 probe were seeded onto each well of a 6-well plate (9.5 cm^2 of growth area) two days before the experiment. Each well contained a total of 2 mL of solution after addition of the H_2O_2 solution. The day of the experiment, cells were imaged in RPMI media. Data points represent the mean ratio ± S.E.M. for 8 different cells and reflects two biological replicates.

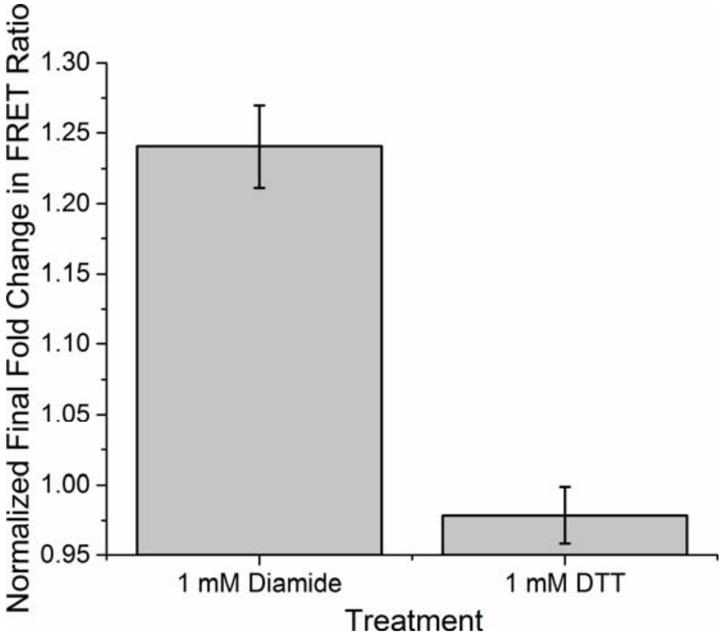

Supplementary Figure 11 | Normalized peak fold change in emission ratio of probe upon stimulation with either 1 mM diamide or 1 mM DTT. Final fold change in signal normalized to signal from sample treated with media only. Data points in represent mean normalized fold change ± S.E.M. for 18 cells over two biological replicates.

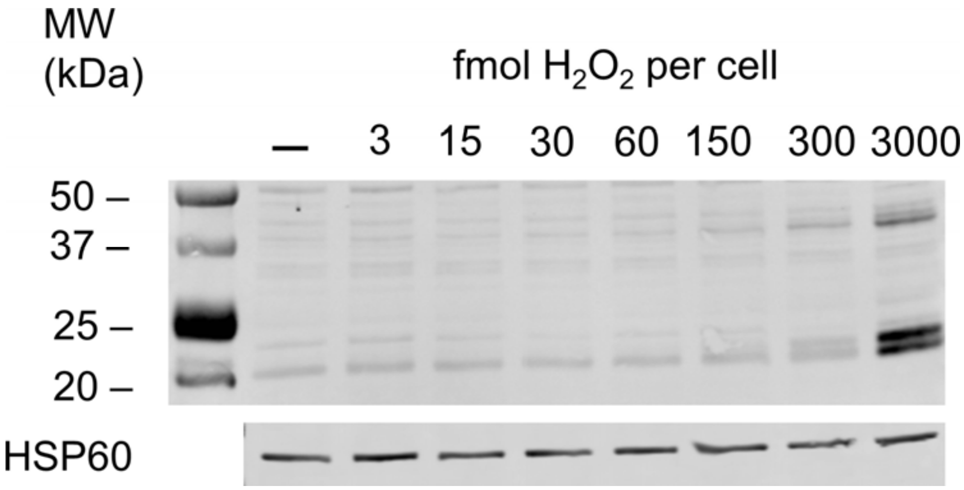

Supplementary Figure 12 | Hyperoxidized Prx western blot of whole cell lysates from HeLa cells stably expressing the Prx2-based probe. Approximately $3.5x10^5$ cells stably expressing the probe were seeded onto a 35mm dish and grown for approximately 24 hours before treatment with H_2O_2. 1mM H_2O_2 used as a positive control. Representative of two western blots.

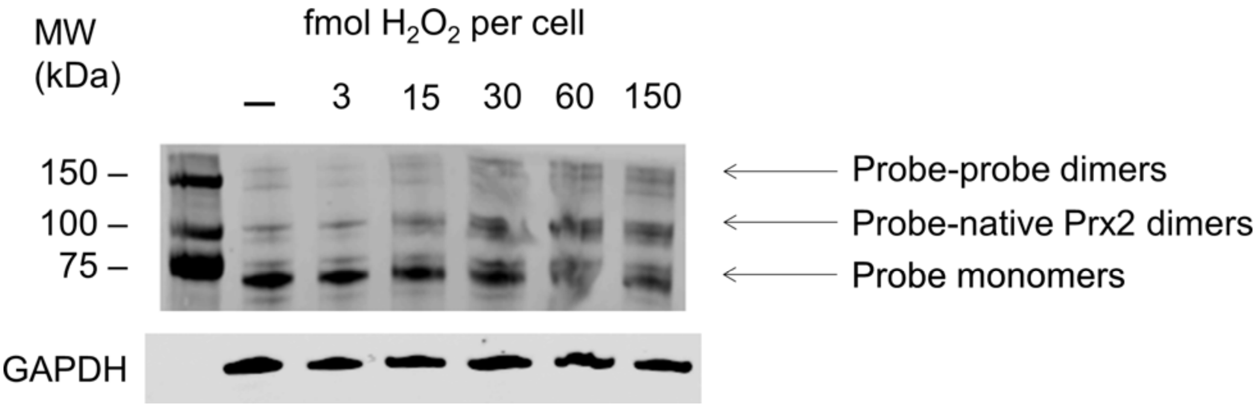

Supplementary Figure 13 | Prx2 western blot of whole cell lysates from HeLa cells stably expressing the Prx2-based probe. Approximately 3.5×10^5 cells stably expressing the probe were seeded onto a 35mm dish and grown for approximately 24 hours before treatment with H_2O_2. Bands at 75 kDa represent the reduced Prx2-based probe monomers, while the bands at approximately 100 kDa and 150 kDa represent the oxidized probe-native Prx2 and probe-probe disulfide linked dimers, respectively. Protein membranes were probed with anti-GFP antibody. Representative of two western blots.

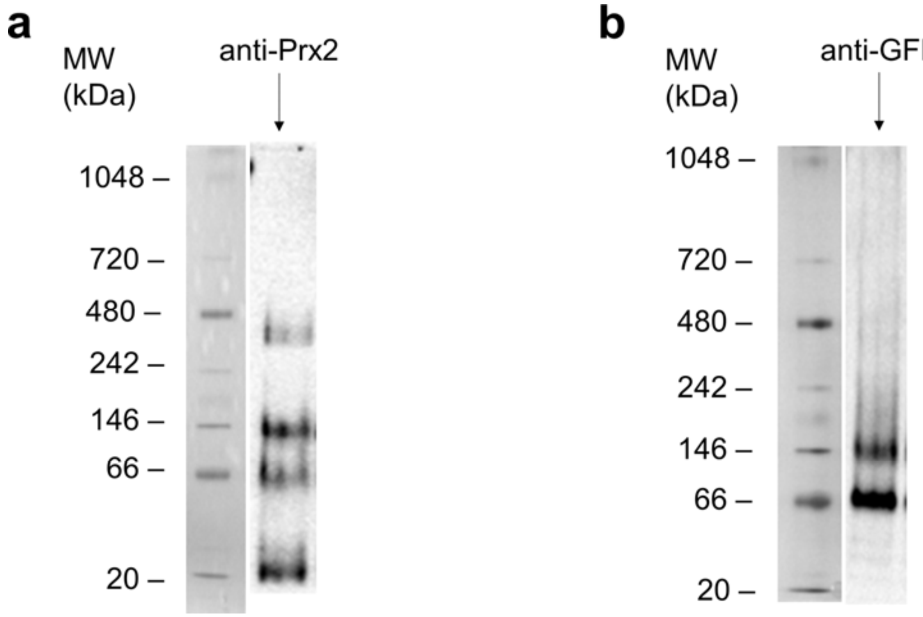

Supplementary Figure 14 | Blue Native PAGE of cytosolic fraction of HeLa cell lysates followed by western blotting for Prx2-based probe molecules. (**a-b**) Approximately $3.5x10^5$ cells stably expressing the probe were seeded onto a 35mm dish and grown for approximately 24 hours before treatment with H_2O_2. Cells were lysed via disruption with glass beads, and the cytosolic fraction of lysates were separated on a bis tris gel, transferred to a PVDF membrane, probed with an anti-Prx2 antibody (**a**) or an anti-GFP antibody (**b**) followed by a secondary antibody conjugated to HRP. Tagged protein on the membrane was detected via treatment with chemiluminescent substrate. Two replicates were done for each blot.

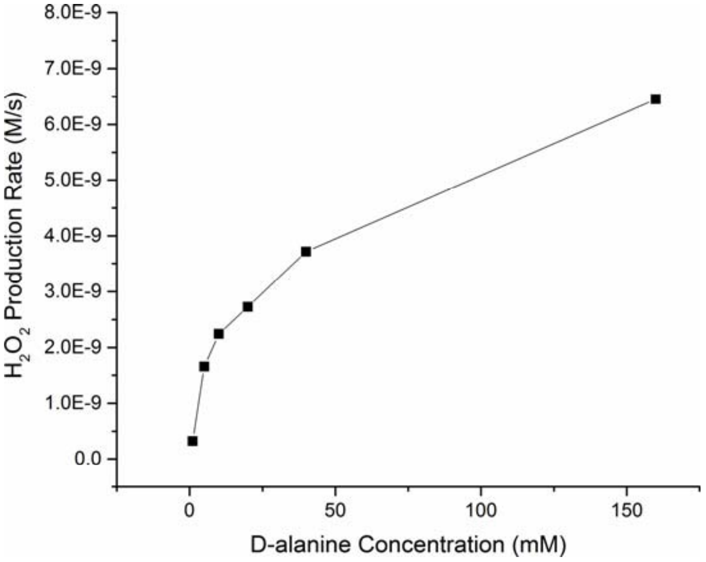

Supplementary Figure 15 | DAAO-mediated-H_2O_2 production rate in HeLa cell lysate. H_2O_2 production in cell lysate for seven different D-alanine concentrations. DAAO was stimulated with indicated concentrations of D-alanine and H_2O_2 production over time was monitored via horseradish peroxidase-amplex red assay (see supplementary methods section for more details). Error bars represent mean production rate ± S.E.M. for two replicates. Michaelis-Menten analysis of the production rate data suggested that the DAAO concentration in the reaction mixture was approximately 18 pM. Based on a lysate dilution factor of 100, a lysate volume of 100 μL, a total number of cells of approximately 7 x 10^5, and a cell volume of approximately 4.2 x 10^{-12} L (based on a sphere with a radius of 10 μm), the DAAO concentration in the cells was calculated to be approximately 60 nM. Since the cytosol does not occupy the total volume of the cell, we expect that the DAAO concentration in the cytosol could be higher than our estimate.

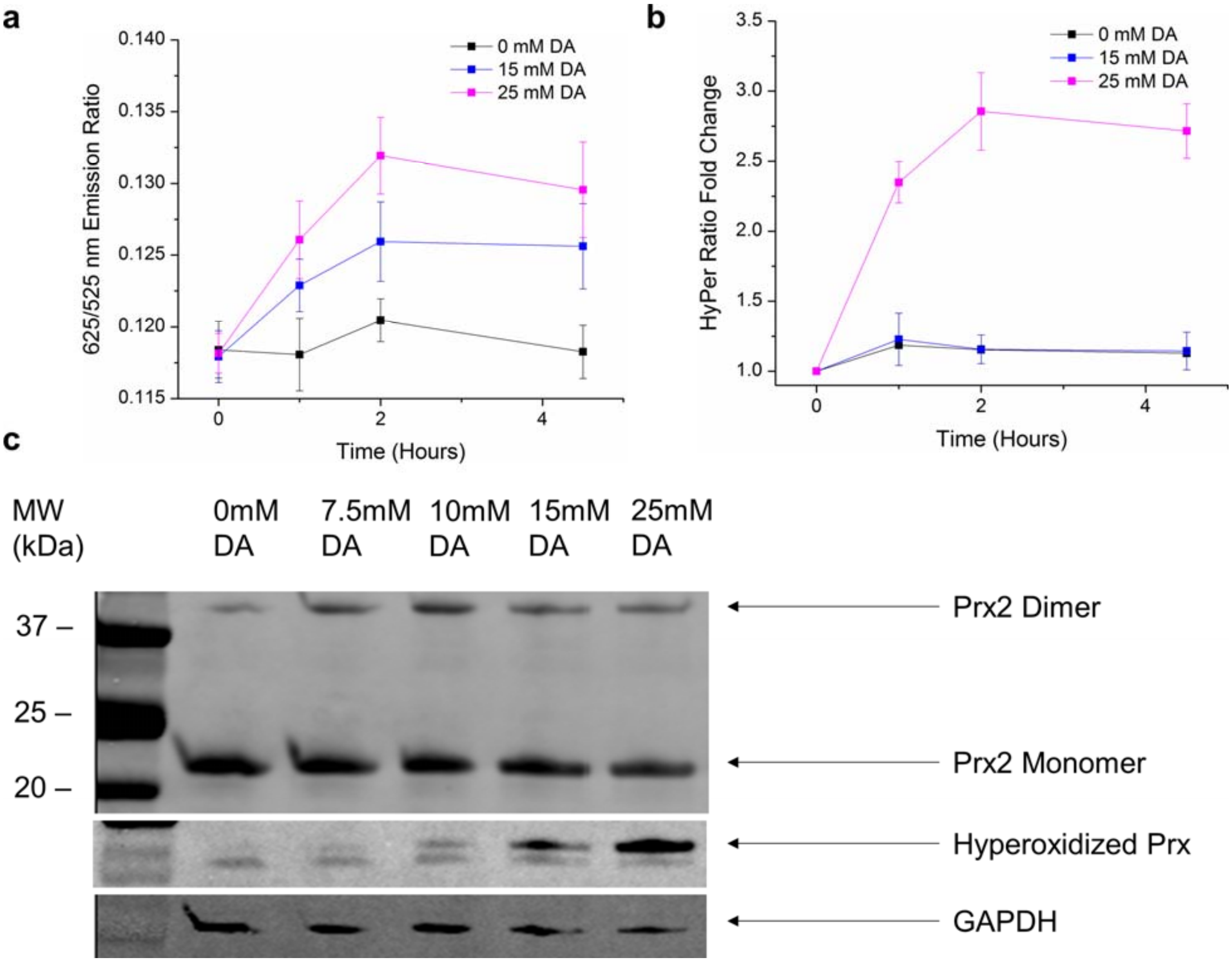

Supplementary Figure 16 | Fluorescent signal from Prx2-based probe and HyPer over time in response to DAAO-mediated intracellular H_2O_2 generation. (**a**) Fluorescent signal from Prx2 based probe over 4.5 hour period upon stimulation with three different concentrations of D-alanine. Data points represent mean emission ratio ± S.E.M. for 18 cells and reflect results from two biological replicates. (**b**) Fluorescent signal from HyPer over 4.5 hour period upon stimulation with three different concentrations of D-alanine. Data points represent mean fold change ± S.E.M. for 18 cells and reflect results from two biological replicates. (**c**) Western blot of whole cell lysates from HeLa cells stably expressing HyPer treated with various amounts of DA.

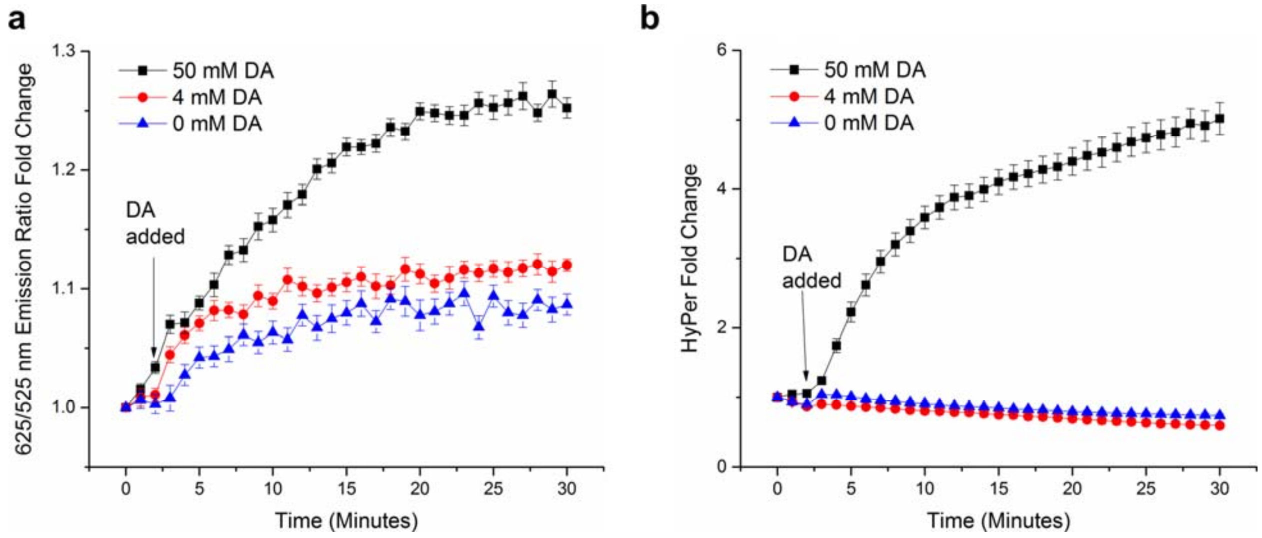

Supplementary Figure 17 | Fluorescent signal from Prx2-based probe and HyPer on the minutes timescale after bolus addition of either D-alanine or medium only to cultures of cells expressing D-amino acid oxidase (**a**) Fluorescent signal from Prx2 based probe over 30 minute period upon stimulation with two different concentrations of D-alanine. Data points represent mean emission ratio fold change ± S.E.M. for 18 cells. (**b**) Fluorescent signal from HyPer over 30 minute period upon stimulation with two different concentrations of D-alanine. Data points represent mean HyPer fold change ± S.E.M. for 18 cells. 50 mM of D-alanine was chosen as a positive control.

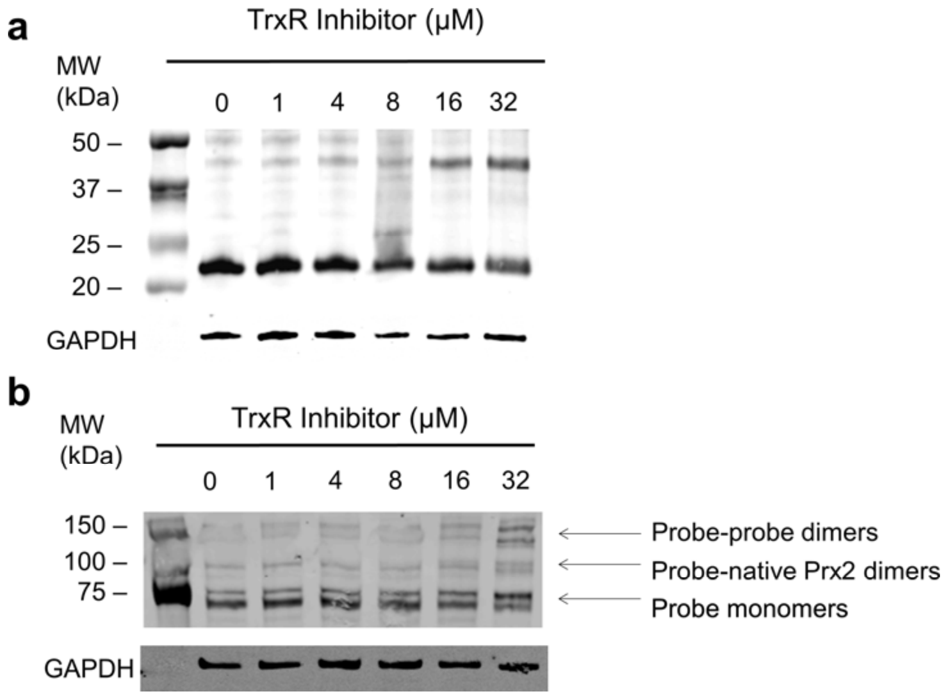

Supplementary Figure 18 | (**a-b**) Western blot of whole cell lysates from HeLa cells stably expressing the Prx2-based probe treated with the thioredoxin reductase inhibitor auranofin for endogenous Prx2 (**a**) and the Prx2-based probe (**b**). Approximately $3.5x10^5$ cells stably expressing the probe were seeded onto a 35mm dish and grown for approximately 24 hours before treatment with Trx inhibitor. Representative of two western blots.

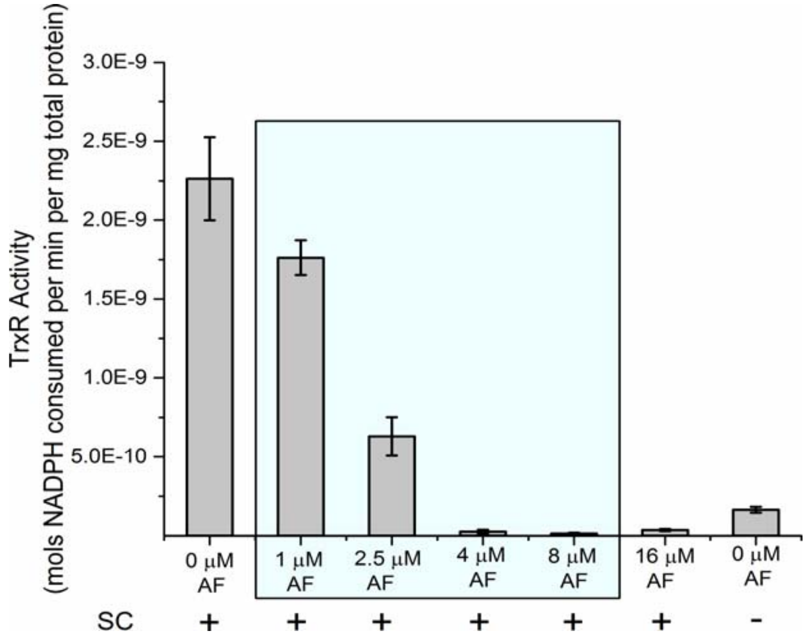

Supplementary Figure 19 | Selenocystine assay for measurement of thioredoxin reductase activity in HeLa cell lysates treated with auranofin. HeLa cell lysates treated with or without auranofin were mixed with solutions containing selenocytsine (SC), a substrate for thioredoxin reductase, and NADPH, a cofactor for thioredoxin reductase. The NADPH absorbance at 340 nm was then measured over a period of 60 minutes. In samples with no auranofin, active thioredoxin reductase reduces SC in the solution, which results in a decrease in the amount of NADPH and the absorbance at 340 nm, whereas in samples with auranofin, inhibited thioredoxin reductase does not reduce SC, which results in less NADPH consumption. Data points represent mean value ± S.E.M. for two biological replicates with two technical replicates each.

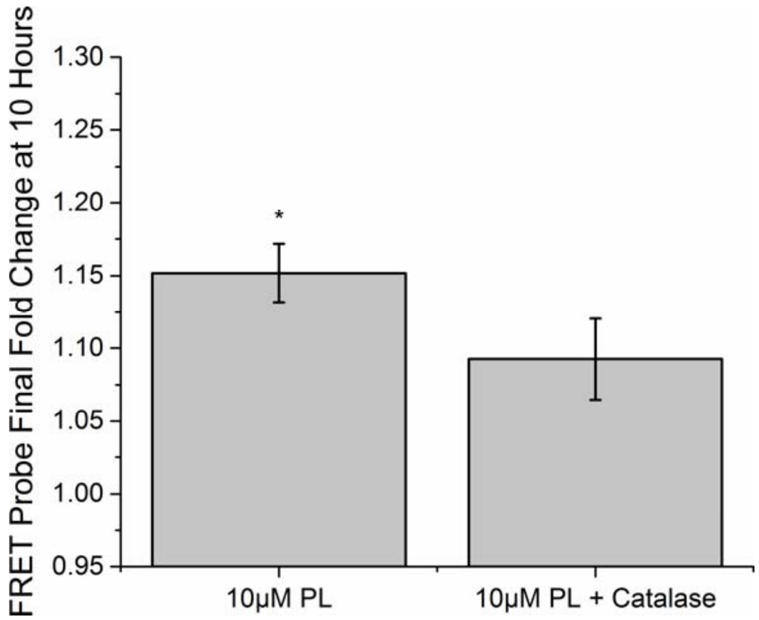

Supplementary Figure 20 | Response of Prx2-based probe to the redox therapeutic compound piperlongumine in HeLa cells with or without additional cytosolic catalase. Plot shows emission ratio fold change at 10 hours for the two different conditions. Data points represent mean fold change ± S.E.M. for 27 HeLa cells. *P<0.10

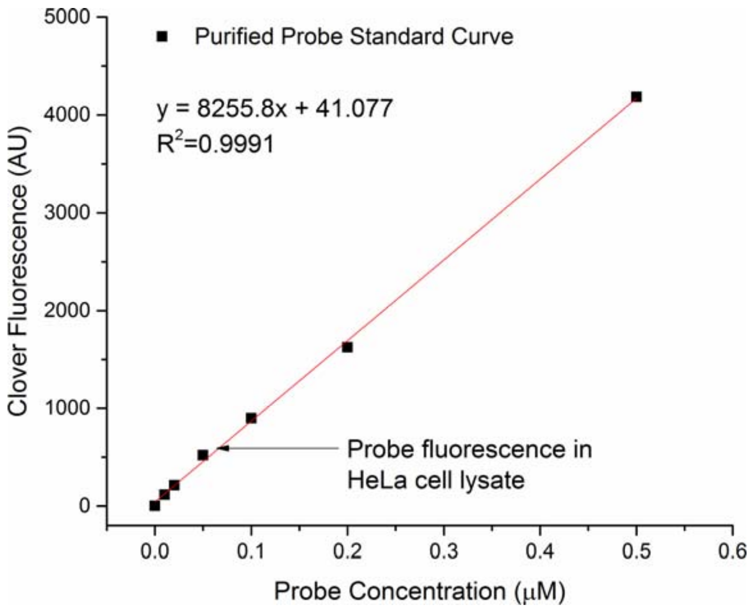

Supplementary Figure 21 | Estimation of intracellular probe concentration from purified protein fluorescence standard curve and lysate probe fluorescence. A standard curve of Clover fluorescence for various concentrations of purified protein in lysis buffer was first constructed. Next, the Clover fluorescence in three separate cell lysate samples was measured and averaged. The probe concentration in the lysate was then estimated with the best-fit equation for the linear regression. Finally, the probe concentration in the cell was estimated based on the total volume of lysate solution, the number of cells used to prepare the lysate, and the volume of the cell. Based on an average lysate fluorescence of 557 ± 85 (n = 3 for three biological replicates), the probe lysate concentration was expected to be approximately 0.062 ± 0.010 μM. Based on a lysate volume of 100 μL, a total number of cells of approximately 1.4 x 10^6, and a cell volume of approximately 4.2 x 10^{-12} L (based on a sphere with a radius of 10 μm), the probe concentration in the cells was calculated to be approximately 1.1 ± 0.2 μM. Since the cytosol does not occupy the total volume of the cell, we expect that the probe concentration in the cytosol could be higher than our estimate, but still on the order of 1 μM.

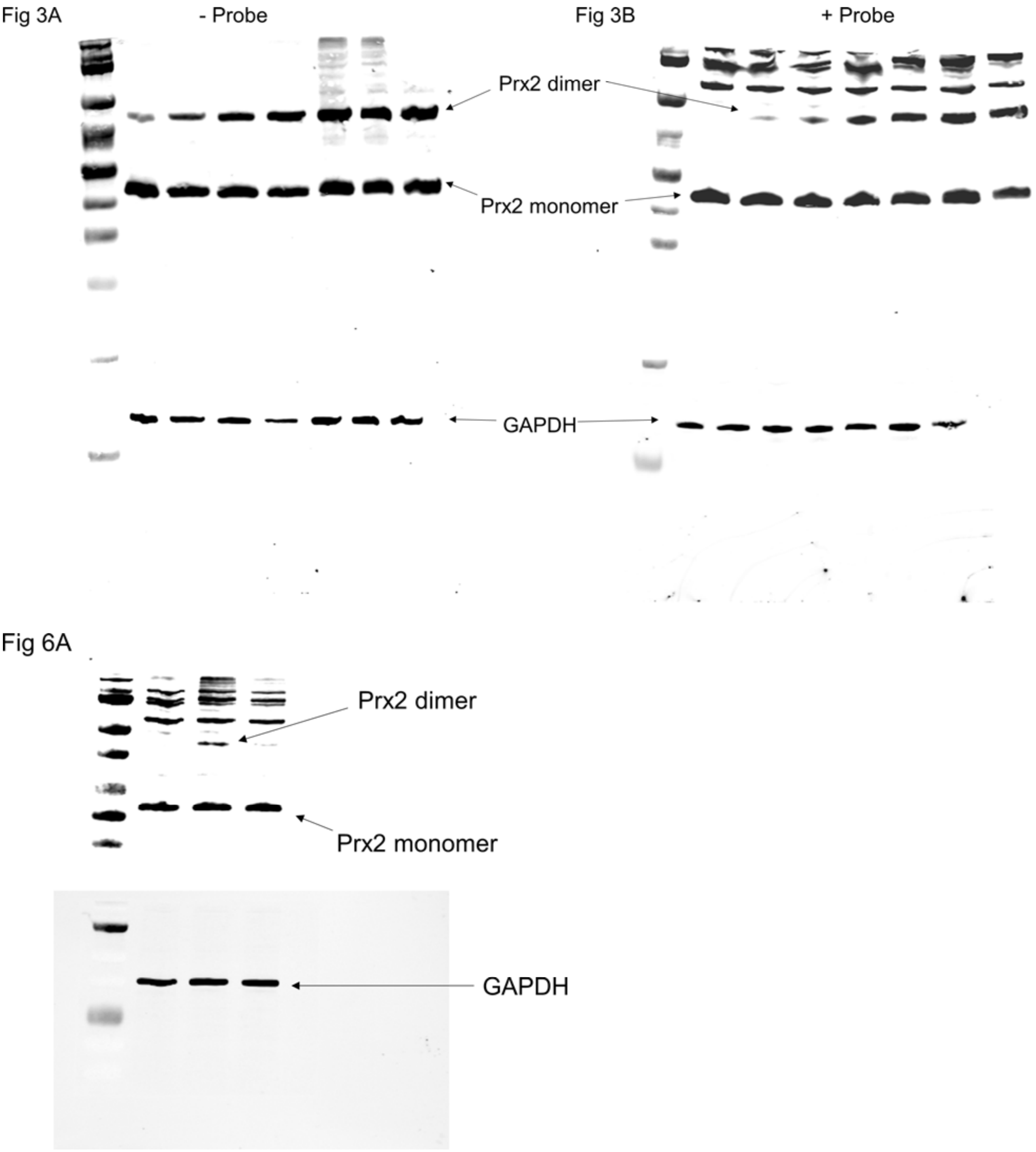

Supplementary Figure 22 | Uncropped versions of western blots in main text. Key bands highlighted for ease of visualization.

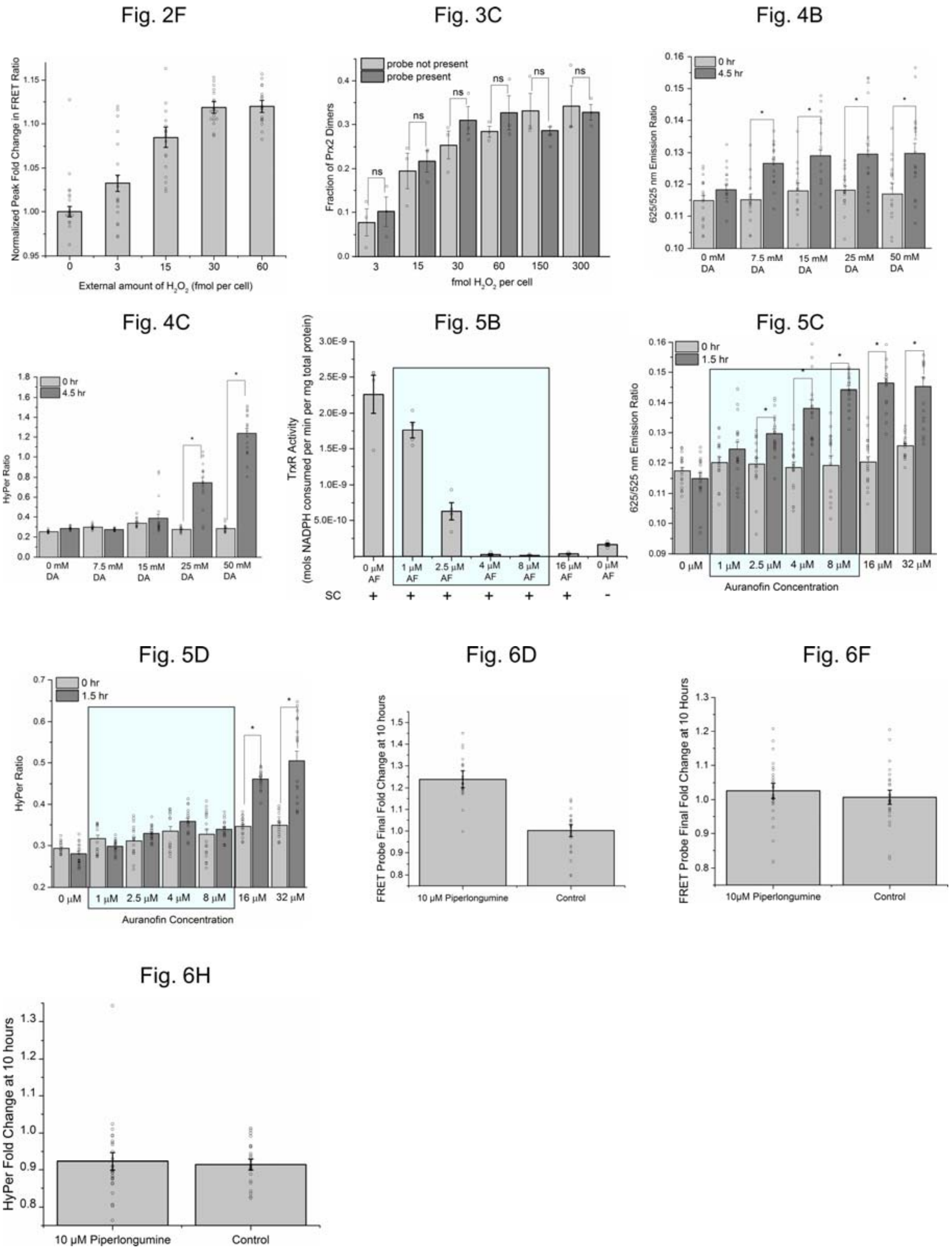

Supplementary Fig. 23 | Bar graphs from main text with dot pots on top of bar graphs to indicate individual data points.

Supplementary Methods

Bacterial expression of FRET probe and purification of fluorescent construct. The gene encoding the fluorescent construct was cloned into the bacterial expression vector pET28b. The gene was inserted into the vector such that it contained a histidine purification tag at the N-terminal of the protein. The vector plus insert was then transformed into BL21 bacterial cells and the bacteria cells were grown to an OD of 0.4. IPTG was then added to the cells at a final concentration of 0.5 mM and the cells were grown for 20 hours at 20°C in order to induce expression of the recombinant protein. After the 20-hour incubation period, the cells were pelleted and lysed via sonication. The filtered cell lysate was then applied to a metal affinity purification column and eluted into buffer contained 50 mM Tris, 300 mM NaCl, and 500 mM imidazole (pH 7.6). The purification elution buffer was then exchanged with 50 mM Tris, 100 mM NaCl, and 10% (v/v) glycerol (pH 7.6) via dialysis. Purified protein aliquots were prepared and frozen at -80°C for future use.

Characterization of purified FRET probe reactivity to various oxidants and buffers. Purified protein was first incubated with 10 mM DTT for 30 minutes at room temperature in order to completely reduce any oxidized protein. DTT was then removed from the protein solution via micro bio-spin P-6 gel columns (Bio-Rad) and the resulting protein solution was placed on ice. Before any oxidants were added to the protein solutions, the baseline signal was acquired in each fluorescent channel. Immediately after baseline signal acquisition, the purified protein was mixed with each oxidant species and the signal in each fluorescent channel was measured again after 4 minutes. For pH sensitivity experiments, the reduced fluorescent construct was first diluted into buffers with pH values in the range of 6.0-8.0. Samples were then

treated with either a control bolus of buffer or 40 μM H_2O_2. For experiments with the peroxynitrite generator SIN1-HCl, measurements were made every two minutes for a total of 30 minutes.

Blue native gel electrophoresis and immunodetection of native proteins Adherent cells were first washed and treated with PBS followed by MMTS as described previously. After incubation with MMTS followed by two PBS washes, cells were detached from the dish with 0.25% Trypsin-EDTA and centrifuged for 5 minutes at 1000 rpm. Cells were then washed with PBS and centrifuged again for 5 minutes at 1000 rpm. Cells were then resuspended in 100 μL of lysis buffer containing 50 mM Tris, 50 mM NaCl, 100 μM EDTA, 10% (v/v) glycerol, and 1x HALT protease inhibitor cocktail at a pH of 7.6. The cells solution was then added to approximately 50 μL of glass beads (Biospec Products). The cell solution with the glass beads was then subjected to disruption via a thermomixer (Eppendorf). Cells were subjected to 4 minutes of disruption at 16000 rpm followed by 1 minute of rest. This step was repeated 5 times and all disruption steps were completed at 4°C. After disruption, the lysed cells were centrifuged for 5 minutes at 16000 g in order to pellet any beads and cell debris. The supernatant of the solutions was then collected and the protein content in the solution was assessed with the BCA assay. 16 μg of cytosolic protein was then loaded into a 3-15% bis tris gel (ThermoFisher Scientific) and subjected to blue native PAGE according to the manufacturer's instructions. After gel electrophoresis, gels were transferred to a PVDF membrane (1 hour at 75V). After transfer, the PVDF membranes were placed in acetic acid for 15 minutes to fix the proteins, washed in deionized water, air-dried, and then placed in methanol to remove the blue coomassie dye. Membranes were then incubated with goat primary antibody for green fluorescent protein (R&D Systems, Catalog # AF4240) (at a

dilution of 1:200) at 4 °C overnight and then with donkey anti-goat antibody conjugated to horseradish peroxidase (Santa Cruz Biotechnology) (at a dilution of 1:1000) for 1 hour at approximately 22 °C. The membranes were then incubated with Pierce ECL western blotting substrate and visualized via a chemiluminescent imager.

Determination of D-amino acid oxidase (DAAO) activity in HeLa cell lysate.

3.5×10^5 Hela cells stably expressing D-amino acid oxidase were seeded per well in a 6-well plate in 2mL of DMEM with 10% FBS. The following day, the cells were lysed with 100µL of 1% Triton-X in PBS with HALT protease inhibitor cocktail (1x). Supernatant was collected, chilled on ice, and tested for an enzymatic activity following the Amplex UltraRed reagent protocol (Thermofisher, Catalog #A36006). The samples were mixed with the Amplex UltraRed reagent working solution with concentration of D-alanine of 160, 80, 40, 20, 10, 5, 1 and 0 mM. Fluorescence was measured using a Tecan M200 plate reader at 1.5 minute intervals for 30 minutes. Excitation and emission wavelengths were set at 490 nm and 585nm, respectively. H_2O_2 production rates over the range of D-alanine concentrations was determined following the manufacturer's protocol. Then, the total enzyme concentration was calculated using the Lineweaver-Burk plot with a turnover rate taken from the literature[3]. The absolute amount of enzyme in the lysate was calculated by multiplying the reaction volume of 100µL and the dilution ratio of 100. To calculate the concentration of DAAO per cell, the absolute amount of enzyme was divided by the total number of cells and then the total volume of cells.

Supplementary References

1. K. Schmidt, W. Desch, P. Klatt, W. R. Kukovetz, B. M. Release of NO from Donor Compounds: A Mathematical Model for Calculation of NO Concentration in the Presence of Oxygen. *Methods Mol. Biol.* **100,** 281–289 (1998).
2. Kavdia, M. & Lewis, R. S. Nitric oxide delivery in stagnant systems via nitric oxide donors: a mathematical model. *Chem. Res. Toxicol.* **16,** 7–14 (2003).
3. L. Pollegioni, B. Langkau, W. Tischer, S. Ghisla, M. Pilone, Kinetic mechanism of D-amino acid oxidases from Rhodotorula gracilis and Trigonopsis variabilis, *J. Biol. Chem.* **268,** 13850–13857 (1993).
