# Supplementary material for: Monitoring the action of redox-directed cancer therapeutics using a human peroxiredoxin-2-based probe
Source: Nat Commun. 2018 Aug 7;9:3145. doi: 10.1038/s41467-018-05557-y (PMC6081480; doi:10.1038/s41467-018-05557-y)
Supplement: Supplementary file 2 — Description of Additional Supplementary Files [file 41467_2018_5557_MOESM2_ESM.pdf]

Description of Additional Supplementary Files

File Name: Supplementary Data 1
Description: Sequences of two plasmids encoding the Prx probe are provided in the accompanying Excel file. Tab 1: Probe_pcDNA3.1+; Tab 2: Probe_pLJM1
